# 34-kDa salivary protein enhances duck Tembusu virus infectivity in the salivary glands of *Aedes albopictus* by modulating the innate immune response

**DOI:** 10.1038/s41598-023-35914-x

**Published:** 2023-06-05

**Authors:** Chalida Sri-in, Aunyaratana Thontiravong, Lyric C. Bartholomay, Wittawat Wechtaisong, Kritsada Thongmeesee, Elizabeth Riana, Sonthaya Tiawsirisup

**Affiliations:** 1 Animal Vector-Borne Disease Research Unit, Veterinary Parasitology Unit, Department of Veterinary Pathology, Faculty of Veterinary Science, Chulalongkorn University, Bangkok, 10330 Thailand; 2 Department of Veterinary Microbiology, Faculty of Veterinary Science, Chulalongkorn University, Bangkok, Thailand; 3 Department of Pathobiological Sciences, School of Veterinary Medicine, University of Wisconsin–Madison, Wisconsin, USA

**Keywords:** Molecular biology, Zoology, Diseases

## Abstract

Duck Tembusu virus (DTMUV) is an important flavivirus that can be transmitted to poultry via *Aedes albopictus* bites. Furthermore, humans residing in the DTMUV epidemic area display activated antiviral immune responses to local DTMUV isolates during the pathogenic invasion, thereby raising the primary concern that this flavivirus may be transmitted to humans via mosquito bites. Therefore, we identified the gene *AALF004421*, which is a homolog of the 34-kDa salivary protein (34 kDa) of *Ae. albopictus* and studied the salivary protein-mediated enhancement of DTMUV infection in *Ae. albopictus* salivary glands. We observed that double-stranded RNA-mediated silencing of the 34 kDa in mosquito salivary glands demonstrated that the silenced 34 kDa impaired DTMUV infectivity, similar to inhibition through serine protease. This impairment occurred as a consequence of triggering the innate immune response function of a macroglobulin complement-related factor (MCR). 34-kDa in the salivary gland which had similar activity as a serine protease, results in the abrogation of antimicrobial peptides production and strong enhance DTMUV replication and transmission. Although the function of the 34 kDa in *Ae. albopictus* is currently unknown; in the present study, we showed that it may have a major role in DTMUV infection in mosquito salivary glands through the suppression of the antiviral immune response in the earliest stages of infection. This finding provides the first identification of a prominently expressed 34 kDa protein in *Ae. albopictus* saliva that could serve as a target for controlling DTMUV replication in mosquito vectors.

## Introduction

Duck Tembusu virus (DTMUV) is a prevalent cause of arthropod-borne viral disease in poultry such as ducks, chickens, geese, and several other avian species. DTMUV is a positive single-stranded RNA virus of approximately 11 kb in length that is classified as a member of the family Flaviviridae and genus *Flavivirus.* Many flaviviruses are prevalent mosquito-borne diseases that annually cause major public health problems, such as West Nile virus (WNV), Dengue virus (DENV), Yellow fever virus (YFV), Zika virus, and Japanese encephalitis virus, in regions with high densities of mosquito vectors^1–3^. Several species of mosquitoes in the genera *Culex* and *Aedes* such as *Culex tritaeniorhynchus*, *Cx. quinquefasciatus, Aedes aegypti,* and *Ae. albopictus* can transmit DTMUV^4–6^. The pathogenicity of DTMUV causing severe illness in mammals, such as BALB/c and Kunming mice, has been investigated in several studies in the past^7–9^. The relevance of DTMUV in humans as natural hosts remains unclear. People, with or without a history of contact with ducks and residing in the DTMUV epidemic area in China and Thailand, produce viral RNA (vRNA) and activate antiviral immune responses to local DTMUV isolates during pathogenic invasion^10,11^. Because *Ae. abopictu*s have circumstances related to humans, there is a primary concern that this flavivirus is possibly transmitted to humans via mosquito biting.

Insects have evolved effective immune systems to defend themselves against pathogen-induced deterioration and caused low significant pathogenesis^12,13^. Because insects lack immunoglobulin-based adaptive immune responses, innate immune responses play a dominant role in detecting and combating microbial infections in insects, and these processes rely on RNA interference (RNAi) and complement activation with cytokine-like responses^14,15^. A complement system is a group of proteins that normally take an inactive form and will be induced further activation by the antigen/antibody complex or immune complex^16^. The complement-like system recognizes flavivirus in *Ae. aegypti* for elimination by macroglobulin complement-related factor (MCR)-mediated responses, which regulate the induction of antimicrobial peptides (AMPs)^17^. Furthermore, it was recently demonstrated that a 34 kDa salivary protein enhances DTMUV infection and transmission by regulating AMPs in the *Ae. aegypti* salivary gland^18^. Therefore, complement-related proteins are also believed to play a crucial role in innate immunity in *Ae. albopictus*.

AMPs comprise a group of short peptides that electrostatically or hydrophobically interact with microbial surfaces to orchestrate their elimination via different mechanisms, including lysis, disruption of proton gradients, or membrane perturbation^19,20^. AMPs play an important role in midgut immunity, which is essential for controlling unexpected microbial overgrowth or opportunistic infections in the gut lumen^21–23^. Most AMPs are induced by gram-negative bacteria via a process controlled by the immune deficiency (Imd) pathway. Contrarily, defensin (DEF), a group of AMP genes, is induced by gram-positive bacteria through a process activated by the Toll pathway. Nonetheless, gambicin (GAM), an AMP gene discovered only in mosquitoes, is jointly regulated by Imd, Toll, and JAK–STAT pathways^24^. Although several studies have examined AMPs in mosquito vectors, the precise regulatory patterns of AMPs in the *Ae. albopictus* salivary gland remains unknown. The variation in the AMP spectrum suggests the existence of different regulatory mechanisms for responding to microbial infections, including DTMUV infection in *Ae. albopictus*.

During the mosquito feeding process, which is the initial step of the transmission cycle, saliva components modulate vertebrate hemostasis, immunity, and inflammation at the biting site. Over recent decades, the capability of *Aedes* mosquito saliva to regulate the host immune response has been illuminated. *Aedes* saliva influences the enhancement of flavivirus transmission, host susceptibility, disease pathogenicity, and viremia levels^25^. Salivary proteins in *Ae. albopictus* are divided into the apyrase, aegyptin, D7, serine protease, and serpin-like protein families, and 34-kDa salivary protein (34 kDa) is grouped into the serine protease family ^26^. Although 34 kDa of *Ae. aegypti* (*AAEL003600*) has been demonstrated to be immunogenic in flavivirus-infected human keratinocytes^27^ and *Ae. aegypti* salivary gland^18^, the effect of the 34 kDa of *Ae. albopictus* (*AALF004421*) on flavivirus is largely unknown. Therefore, this is possibly the first study to examine the relationship of the 34 kDa with the antiviral immune response to a flavivirus, DTMUV, infection in the *Ae. albopictus* salivary gland. Considering the data obtained in the present study, we propose the existence of a viral enhancement pathway that supports viral replication and transmission by disrupting AMPs in response to DTMUV infection in the *Ae. albopictus* salivary gland.

## Results

### Phylogenetic analysis of 34-kDa salivary protein in *Ae. albopictus*

**Figure 1:**
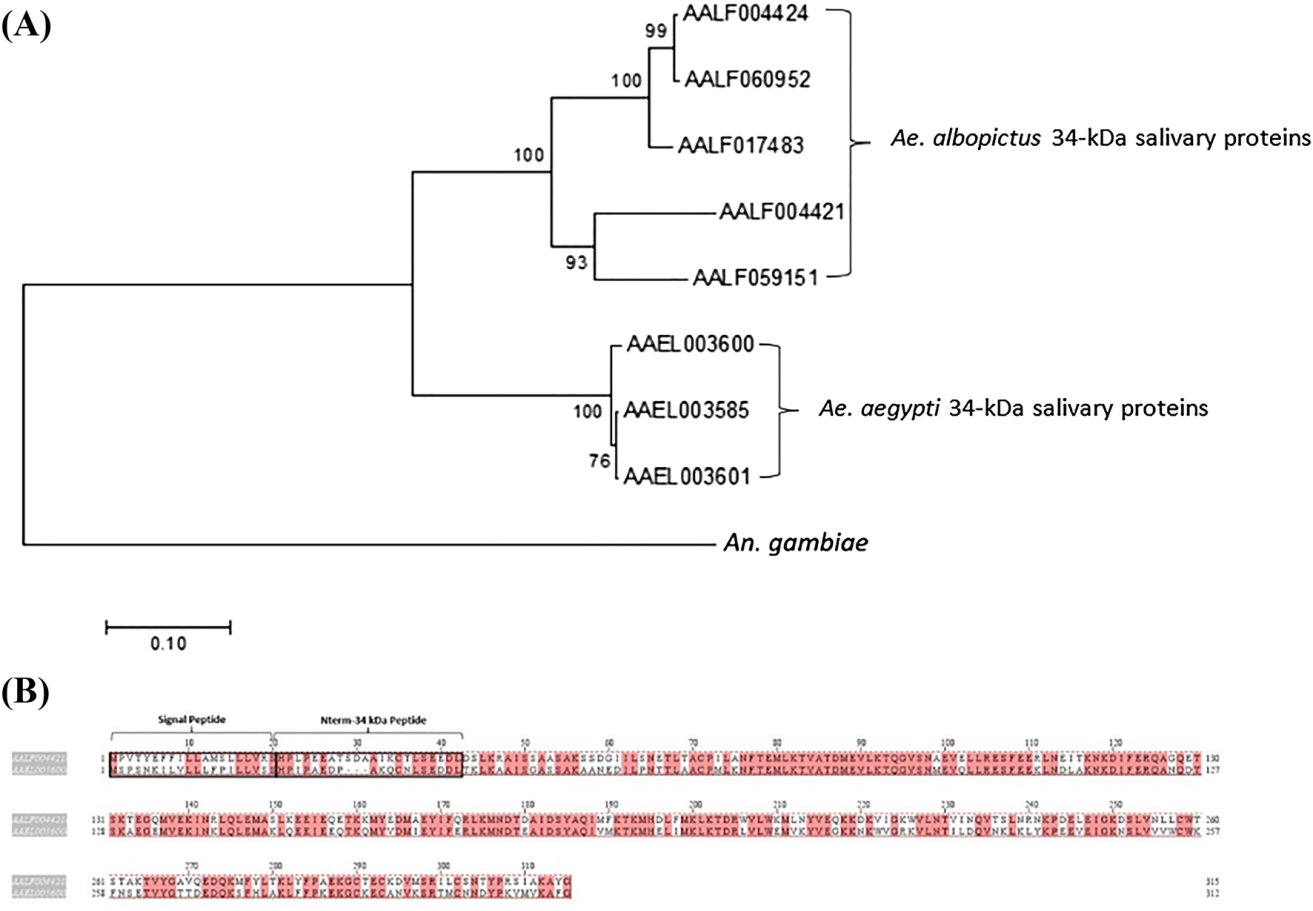
Phylogram showing the bootstrap values compare the 34-kDa salivary proteins from *Ae. aegypti* and *Ae. albopictus*, and using *An. gambiae* sequences as an outgroup. (**A**) Phylogram showing the bootstrap values. (**B**) Clustal alignment.

Using the *Anopheles* sequence as an outgroup, the phylogram shows two clusters with strong bootstrap support. Within the *Aedes* genus, the *Ae. albopictus* and *Ae. aegypti* homologs are distinctly grouped, as expected from these two mosquitoes of the same genus; however, they are clearly separated into different species (Fig. 1A). Among them, the putative 34 kDa secreted salivary protein appeared specific to the *Aedes* genus. Therefore, the N-terminal extremity peptide of the 34 kDa protein (Nterm-34 kDa peptide) appeared to be an interesting candidate for validation as a biomarker specific to *Ae. aegypti* bites^28^. Amino acid sequences of the putative 34 kDa secreted salivary protein of *Ae. aegypti* (*AAEL003600*) and the putative salivary protein 34 kDa of *Ae. albopitus* (*AALF004421*) are presented. Sequences of the Nterm-34 kDa peptide and the signal peptide (SP) are indicated in square frames (Fig. 1B). The alignment of the 34 kDa sequences from *Ae. aegypti* with *Ae. albopictus* indicated that—with the exception of the Nterm-34 kDa peptide and SP sequences—*AALF004421* resembles *AAEL003600*, suggesting the possible conserved sites of these proteins (Fig. 1B). Because *AALF004421* was specified as a putative salivary protein 34 kDa of *Ae. albopictus*^29^, we hence used this gene throughout the present study.

### Phylogenetic analysis of MCR in *Ae. albopictus*

**Figure 2:**
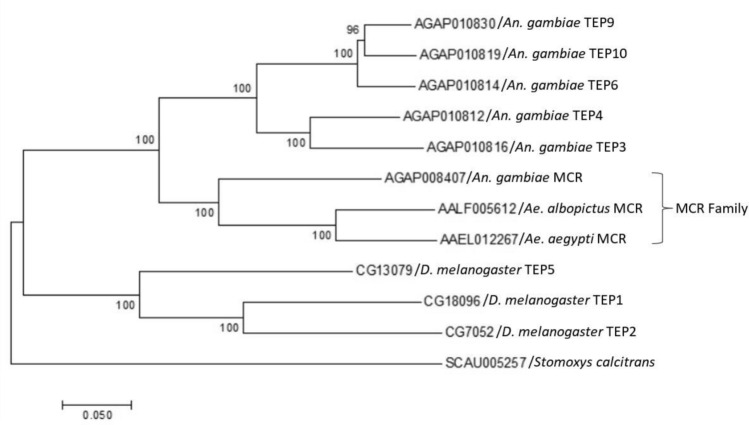
Comparison of the iTEPs from *Ae. albopictus, Ae. aegypti*, *An. gambieae*, and *D. melanogaster*, using *Stomoxys calcitrans* sequence as an outgroup.

Thioester-containing proteins (TEPs), which are crucial components of the innate immune systems in vertebrates and invertebrates, can be divided into three families as follows: insect TEPs (iTEPs), alpha-2-macroglobulins (A2Ms), and C3/C4/C5 complement factors^30^. MCR belongs to the iTEPs family, which is thought to play a key role in complement-like system functions as an antiviral mechanism in arthropods. Therefore, we analyzed the phylogenetic relationships of iTEP proteins among *Ae. albopictus*, *Ae. aegypti*, *Anopheles gambieae*, and *Drosophila melanogaster*, using the *Stomoxys calcitrans* sequence as an outgroup. The results showed that the MCRs cluster along a single branch, which characterize them as a subfamily of iTEPs (Fig. 2). Furthermore, *AALF005612* is grouped with *Ae. aegypti* MCR (*AAEL012267*), indicating that *AALF005612* is an *Ae. albopictus* MCR homolog (Fig. 2). Additionally, recent studies have reported that *AAEL012267*, known previously as *Ae. aegypti* TEP1 or *Ae. aegypti* MCR, has a potential role in combating flavivirus infection of *Ae. aegypti* ^17,31^. However, information related to the response of *Ae. albopictus* MCR to flavivirus infection is lacking. Therefore, we have focused on *AALF005612* as the *Ae. albopictus* MCR and hypothesized that it may be a crucial factor in DTMUV infection in *Ae. albopictus.*

### Expression of 34-kDa salivary protein and MCR in DTMUV-infected salivary glands of *Ae. albopictus*

**Figure 3:**
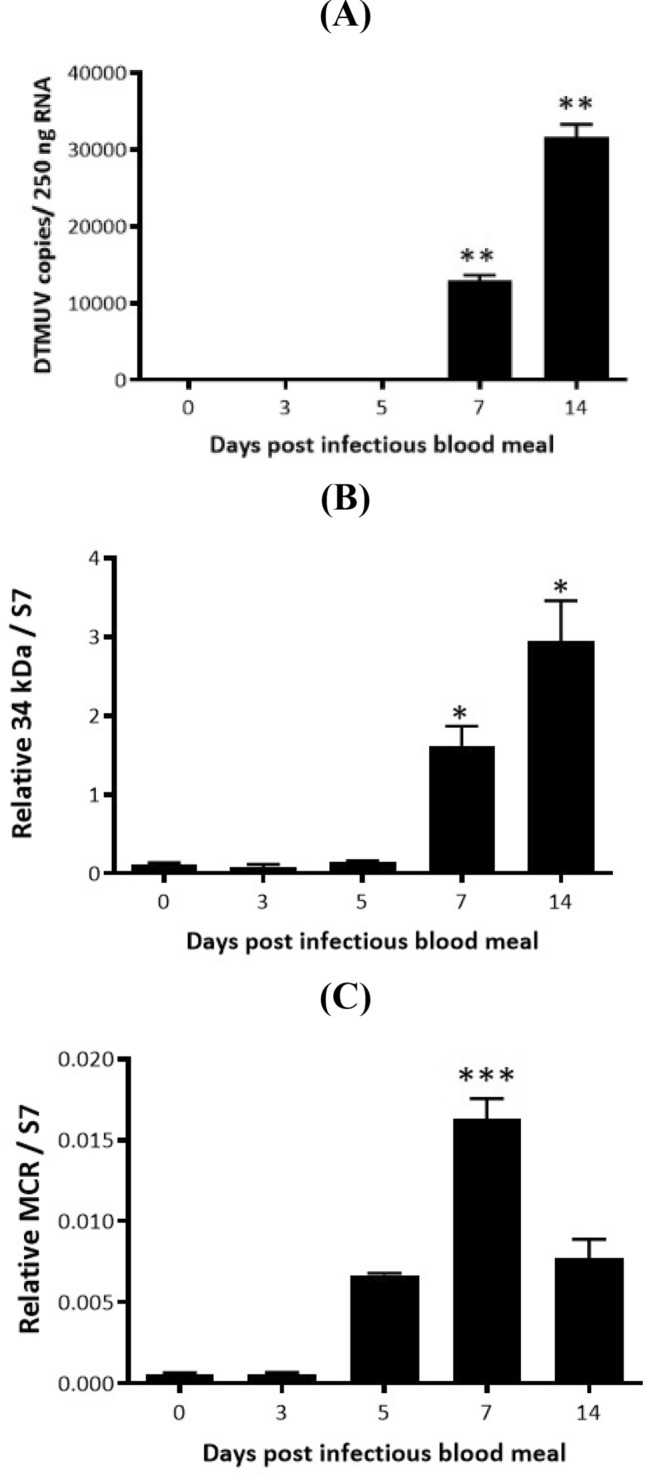
Expression of 34-kDa salivary protein (34 kDa) and macroglobulin complement-related factor (MCR) in the duck Tembusu virus (DTMUV)-infected salivary gland of *Aedes albopictus*. Mosquitoes were given an infectious blood meal. The total RNA of salivary glands was collected (*n* = 50 for each experiment) on days 0, 3, 5, 7, and 14 after feeding. Real-time quantitative polymerase chain reaction was performed to investigate the expression of the 34 kDa and MCR genes in salivary glands following DTMUV infection. (**A**) Viral load in the salivary glands was determined, and the results are presented as the number of DTMUV genome copies per 250 ng of RNA. The relative numbers of 34 kDa (**B**) and MCR (**C**) copies were quantified and normalized against that of S7 ribosomal protein. The results represent three independent experiments, and the data are presented as the mean ± SEM. (*) indicates statistically significant differences between day 0 and each time point (*p < 0.05, **p < 0.01, ***p < 0.001, and ****p < 0.0001; unpaired* t*-test).

To verify the peak expression of the innate immune response throughout DTMUV infection in *Ae. albopictus* salivary glands, the gene transcripts were compared at various time points between days 0 and 14 after infectious blood meal feeding. The kinetics of DTMUV infection in the salivary glands of *Ae. albopictus* at different times was monitored using Real-time quantitative PCR (RT-qPCR). Although the presence of viral transcripts could not be detected in salivary glands at early time points, the viral transcript levels increased from day 7 to day 14 after infectious blood meal feeding (Fig. 3A). The results illustrated that the 34 kDa was highly expressed following DTMUV infection (Fig. 3B). However, the highest *MCR* mRNA copy number was detected at day 7 after infectious blood meal feeding, which is an early time point after salivary glands were infected (Fig. 3C). This indicates that the complement system is activated during the early phase of infection. Owing to the fact that MCR was most strongly expressed and the viral transcript levels were detected from day 7 after infectious blood meal feeding, the salivary glands were harvested on this day for further experimentation.

### Silencing of 34-kDa salivary protein suppresses DTMUV replication in salivary glands and reduces the viral load in the saliva of *Ae. albopictus*

**Figure 4:**
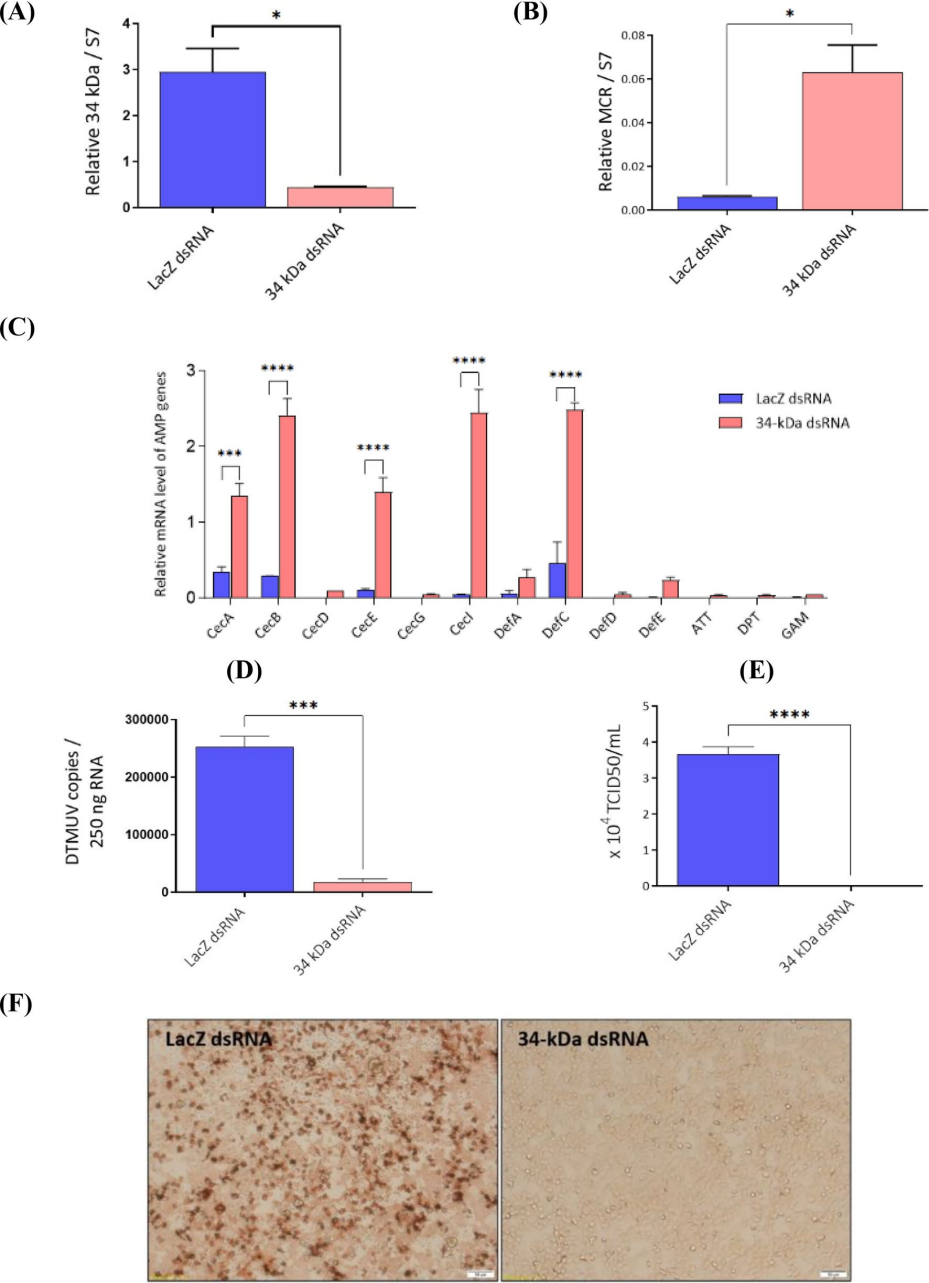
Silencing of 34-kDa salivary protein (34 kDa) reduces duck Tembusu virus (DTMUV) replication in salivary glands and the viral burden in the saliva of *Aedes albopictus*. Mosquitoes were intrathoracically injected with double-stranded RNA (dsRNA) targeting 34 kDa or LacZ (control). Three days later, mosquitoes were orally infected with DTMUV. Total RNA was extracted from the salivary glands of 34 kDa- or LacZ-silenced (*n* = 50 for each treatment) mosquitoes at 7 days post-infectious blood meal (dpb). (**A**) Transcripts of 34 kDa, (**B**) macroglobulin complement-related factor (*MCR*) and (**C**) antimicrobial peptides (*AMPs*) in the salivary glands were determined via real-time quantitative polymerase chain reaction (RT-qPCR), and the relative values were normalized against that of S7 ribosomal protein. (**D**) Viral load in the salivary glands was determined by RT-qPCR and presented as the number of DTMUV genome copies per 250 ng of RNA. (**E**) 50% tissue culture infectious doses (TCID_50_/mL) of saliva were compared between LacZ-silenced control and 34 kDa-silenced mosquitoes at 7 dpb. (**F**) BHK-21 cells were inoculated with saliva (200 µg/mL) from LacZ or 34 kDa dsRNA mosquitoes at 7 dpb to quantify the viable virus content. The results were pooled from three independent experiments, and the data are presented as the mean ± SEM. (*) indicates statistically significant differences between LacZ-silenced control and 34 kDa-silenced mosquitoes (*p < 0.05, **p < 0.01, ***p < 0.001, and ****p < 0.0001; unpaired* t*-test).

To elucidate the relationship between the 34 kDa and MCR that subsequently influences DTMUV infection in salivary glands, female mosquitoes were intrathoracically injected with dsRNA targeting the salivary protein or dsLacZ (control). The mosquitoes were then orally infected with DTMUV on day 3 after gene silencing. The experiment revealed that the 34 kDa was silenced by RNAi, and the gene expression was significantly different from that in the control (Fig. 4A). *MCR* mRNA levels were higher in salivary glands after 34 kDa was silenced (Fig. 4B). Furthermore, owing to the fact that MCR influences antiviral factors, AMPs, which restrict flavivirus infection in mosquitoes, we hypothesized that 34-kDa salivary protein and serine protease enhance DTMUV infection by regulating MCR to inhibit innate immune responses. Therefore, we examined the expression of thirteen AMPs, including four DEF genes, six cecropin (CEC) genes, and one gene in the attacin (ATT), diptericin (DPT), and GAM families, in response to DTMUV infection in *Ae. albopictus* salivary glands. To elucidate whether MCR regulates AMPs as mediated by the 34 kDa, we examined whether the salivary protein could impair AMPs activity in the salivary glands. The results showed that the mRNA expression of five AMPs, namely CECA, CECB, CECE, CECI, and DEFC, was significantly enhanced when the 34 kDa was silenced in DTMUV-infected salivary glands (Fig. 4C). Notably, the viral copy number in 34 kDa-silenced mosquitoes was significantly lower than that in the control group (Fig. 4D). Similarly, the viral titers in saliva from 34 kDa-silenced mosquitoes were significantly lower than from LacZ-silenced mosquitoes (Fig. 4E, data for all replicates performed in the supplementary information file), which was clearly shown in the infected BHK-21 cells after exposure to DTMUV-infected saliva from LacZ-silenced mosquitoes (Fig. 4F). These results demonstrated that 34 kDa silencing suppresses DTMUV replication in salivary glands and mitigates the viral load in the saliva of *Ae. albopictus*.

### Inhibition of serine protease suppresses DTMUV replication in salivary glands and reduces the viral burden in the saliva of *Ae. albopictus*

**Figure 5:**
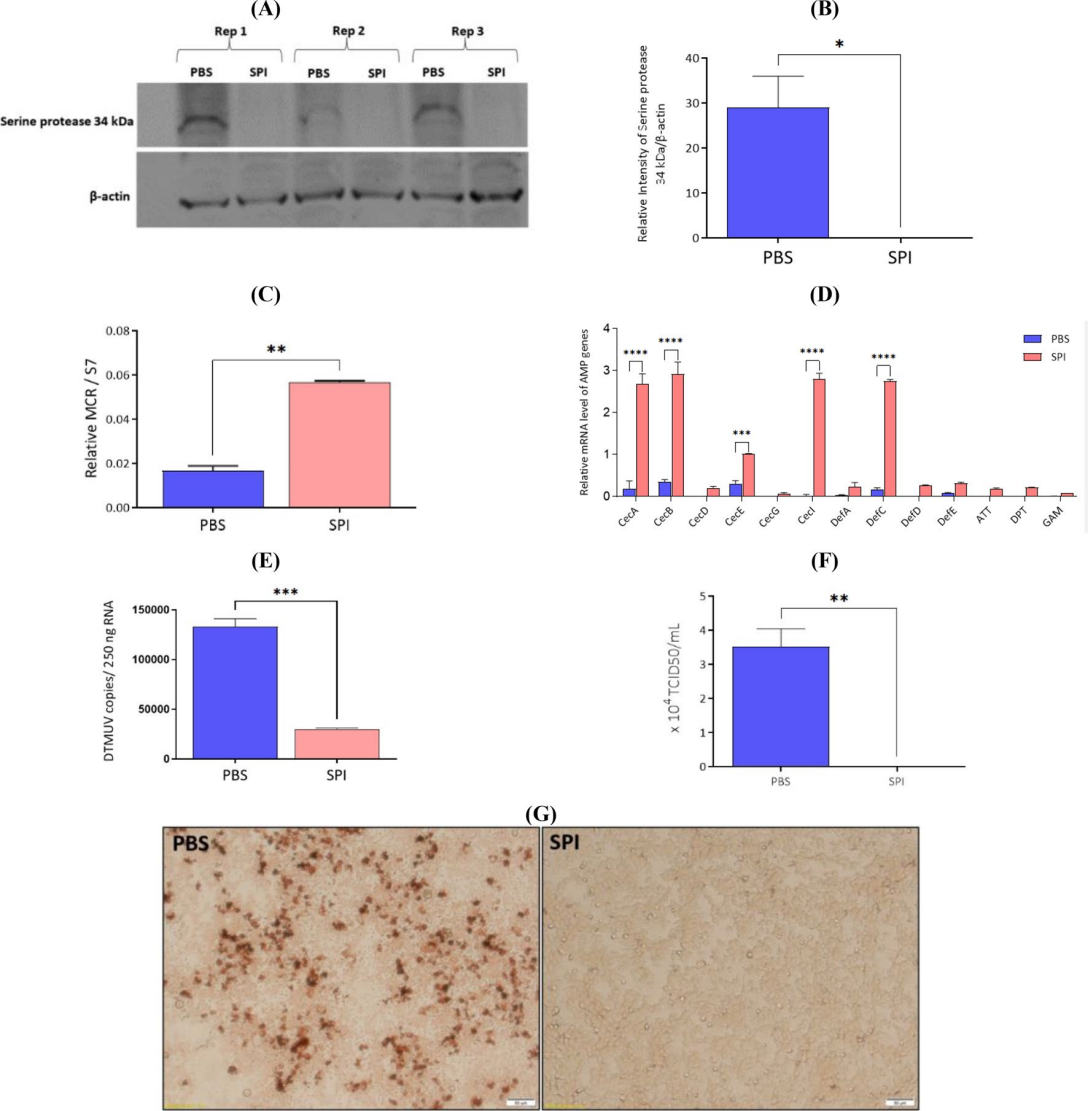
Inhibition of serine protease reduces duck Tembusu virus (DTMUV) replication in salivary glands and the viral burden in the saliva of *Aedes albopictus*. Mosquitoes were intrathoracically injected with a serine protease inhibitor (SPI) or PBS-treated control. Three days later, the mosquitoes were orally infected with DTMUV. (**A**) Salivary glands were collected from SPI and PBS (*n* = 50 for each treatment) mosquitoes at 7 days post-infectious blood meal feeding (dpb) and subjected to Western blotting. The experiment was performed with three replicates. (**B**) The intensity of serine protease relative to β-actin was measured using the ImageJ program. (**C**) Macroglobulin complement-related factor (*MCR*) and (**D**) antimicrobial peptides (*AMPs*) expression in the salivary glands from SPI and PBS mosquitoes were determined on 7 dpb by real-time quantitative polymerase chain reaction (RT-qPCR), and the relative values were normalized against that of S7 ribosomal protein. (**E**) The viral load in the salivary glands was determined using RT-qPCR and presented as the number of DTMUV genome copies per 250 ng of RNA. (**F**) 50% tissue culture infectious doses (TCID_50_/mL) of saliva were compared between SPI and PBS mosquitoes at 7 dpb. (**G**) BHK-21 cells were inoculated with saliva (200 µg/mL) from PBS or SPI mosquitoes at 7 dpb to quantify the viable virus content. These results were pooled from three independent experiments, and data are presented as the mean ± SEM. (*) indicates statistically significant differences between the PBS control and SPI groups (*p < 0.05, **p < 0.01, ***p < 0.001, and ****p < 0.0001; unpaired *t*-test).

Serine protease, one of several subclasses of proteases, is characterized by a serine residue at the active center of the protease. Mosquito saliva serine protease enhances the dissemination of flaviviruses, including DENV and WNV, into mice and NIH 3T3 cells^32^. We speculated that 34 kDa salivary protein serves as a mosquito saliva serine protease that enhances DTMUV infection in salivary glands. The mosquitoes were intrathoracically injected with a serine protease inhibitor (SPI) or PBS-treated control. The inhibition of serine protease after Pefabloc SC treatment was confirmed via the Western blot test (Fig. 5A, original images of full-length blots for all replicates performed in the supplementary information file), and the relative intensity was measured using ImageJ (Fig. 5B). The results of these analyses revealed that no serine protease with a molecular weight of 34 kDa was observed in the mosquitoes after SPI treatment (Figs. 5A and B). *MCR* mRNA data indicated that salivary glands expressing normal serine protease levels had significantly lower *MCR* mRNA levels than those in which serine protease was inhibited (Fig. 5C). Similarly, to elucidate whether serine protease with a molecular weight of 34 kDa enhanced DTMUV infection by regulating AMPs, we determined whether serine protease could block AMP activity in the salivary glands. The results demonstrated that the mRNA expression of five AMPs—i.e., CECA, CECB, CECE, CECI, and DEFC—was significantly enhanced when serine protease was inhibited in DTMUV-infected salivary glands (Fig. 5D). These assessments revealed that silencing the 34 kDa had the same effect on AMP abundance as inhibition of serine protease. In accordance with the outcomes of 34 kDa silencing, mosquitoes expressing serine protease (PBS-treated control mosquitoes) showed a significantly greater number of viral copies in the salivary glands than those lacking serine protease (SPI-treated mosquitoes) (Fig. 5E). Similarly, saliva from PBS-treated control mosquitoes had significantly higher viral titers than those from SPI-treated mosquitoes (Fig. 5F, data for all replicates performed in the supplementary information file), which was showed in the infected BHK-21 cells after treatment with DTMUV-infected saliva from PBS-treated control mosquitoes (Fig. 5G). These results demonstrate that inhibition of serine protease suppresses DTMUV replication in the salivary glands and reduces the viral load in *Ae. albopictus* saliva.

### AMPs directly affected DTMUV infection in *Aedes albopictus*

**Figure 6:**
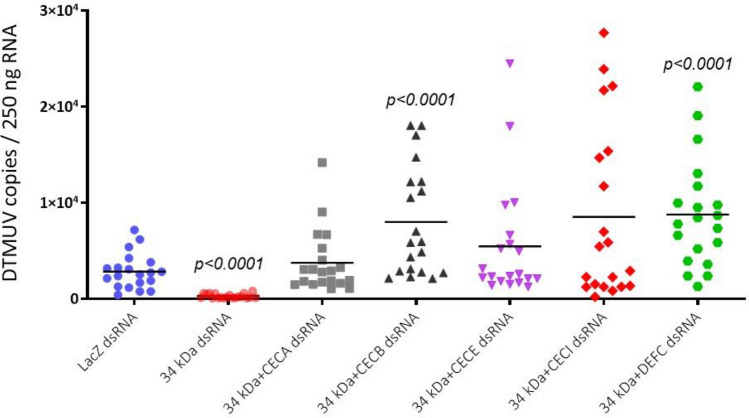
Antimicrobial peptides (AMPs) directly affected duck Tembusu virus (DTMUV) infection in *Aedes albopictus*. In mosquitoes, the individual AMP genes (CECA, CECB, CECE, CECI, and DEFC) were simultaneously silenced with the 34 kDa via the intrathoracic injection of double-stranded RNA (dsRNA). The control mosquitoes were intrathoracically injected with dsRNA targeting LacZ. Three days later, the mosquitoes were inoculated with DTMUV. Total RNA from the mosquito’s whole body was collected on day 7 post-infection. The viral load was assessed by real-time quantitative polymerase chain reaction, and the data were presented as the number of DTMUV genome copies per 250 ng of RNA. The result is representative of two independent experiments. One dot represents an individual mosquito, and the horizontal line represents the mean of the results. Statistically significant differences between the control and experimental mosquitoes were analyzed using an unpaired *t*-test. *P* values less than 0.05 were considered significant.

Numerous types of AMPs exert antiviral activity against flaviviral infections, possibly in a manner similar to their antibacterial activity. Induction of AMP-mediated responses to viral infections in an early phase of invasion is one of the crucial activities against many types of flavivirus in mosquitoes^23,33,34^. Five AMPs, including CECA, CECB, CECE, CECI, and DEFC, were likely regulated by the 34 kDa salivary protein and serine protease. We subsequently examined whether the 34 kDa or AMP encoding genes directly interacted with DTMUV infection by simultaneously silencing the individual AMPs gene (CECA, CECB, CECE, CECI, and DEFC) with the 34 kDa using dsRNA in *Ae. albopictus*. The gene expression of 34 kDa and individual AMPs was significantly decreased after simultaneous treatment with dsRNA. Compared with the findings for LacZ-treated control mosquitoes, as in the previous results, a significant decrease in vRNA replication was observed in mosquitoes after silencing of the 34 kDa alone (Fig. 6). In contrast, vRNA replications tended to increase with simultaneous silencing of the 34 kDa with the individual AMPs gene; in particular, viral burdens were significantly increased in the mosquitoes in which CECB or DEFC was silenced (Fig. 6), suggesting that the 34 kDa did not directly affect DTMUV replication, although AMPs did have an influence. These results indicated that the function of the 34 kDa might be associated with the immune signaling pathways that regulate AMP expression and subsequently facilitate DTMUV infection in *Ae. albopictus*.

## Discussion

We identified that the 34-kDa salivary protein, an exclusive component of the salivary glands of female *Aedes* mosquitoes^29^, was an important protein that enhances the infectivity of flaviviruses such as DENV and DTMUV^18,27,35^. The 34 kDa was reported to inhibit the expression of AMPs following flavivirus infection in human cells and *Ae. aegypti*^18,27,36^. Following its ability to inhibit AMPs, the protein was also capable of inhibiting the expression of IRF3, IRF7, and type I IFN in human keratinocytes^27^. The 34 kDa might correspond to serine protease because the secreted mature serine proteases with varying predicted molecular weights of 28–43 kDa were identified in the sialotranscriptome of the female *Aedes* mosquito^37^. Serine protease is relevant to various physiological activities, such as immunity, coagulation, degradation, and food digestion^38^. Position-specific iterative basic local alignment search tool for the transcript of the 34 kDa protein family against the non-redundant protein database revealed cytoskeleton proteins attributable to the presence of repeated charges that indicated the 34 kDa is probably correlated with adhesion phenomena^37^. Certain types of this enzyme, such as CLIP domain serine protease, are specifically expressed in the female *An. gambiae* sialotranscriptomes, and they resemble prophenoloxidase activators, indicating that certain types of this enzyme alleviate inflammation or activate anti-inflammatory pathways including the complement control protein pathway^37^. Therefore, we hypothesized that 34 kDa is a cofactor rather than the sole effector involved in salivary protein-mediated flavivirus infectivity enhancement.

A serine protease inhibitor was used to irreversibly inhibit the active center of serine protease by forming covalent sulfonyl derivatives of the serine residue, and dsRNA was used to silence the 34-kDa gene by inhibiting and degrading target RNA. The weak expression of the 34 kDa in salivary glands in the absence of DTMUV indicated that the protein was induced via viral infection. dsRNA-mediated silencing of 34 kDa or serine protease inhibition in female *Ae. albopictus* mosquitoes limited viral expression in the salivary gland. Interestingly, silencing the 34 kDa salivary protein or blocking serine protease restricted DTMUV replication affected the increase in *Ae. albopictus* MCR expression. In this study, we demonstrated that 34 kDa and serine protease played a crucial role in supporting DTMUV infection in *Ae. albopictus* that may interact with MCR. To further explore the essential roles in the 34 kDa-based enhancement of viral infectivity, we identified antiviral immune factors such as AMPs and investigated their role in DTMUV infection in *Ae. albopictus* salivary glands. From the data obtained in the present study, we observed that the 34 kDa did not directly interact with the virus. However, its enhancing viral effect was possibly caused by limiting AMP expression following virus invasion.

Although we did not prove that the mosquitoes were bacteria-free, all mosquitoes we used to treat dsLacZ (control) and ds34-kDa were under the same conditions. Since the AMP expression is more related to bacterial infection than arboviral infection, the mosquitoes were treated with antibiotic–antimycotic through viral infectious blood meal to prevent bacterial-induced AMP expression. The study results of post-viral infection indicated statistically significant differences between these two groups of control and 34 kDa-silenced mosquitoes, thereby this study demonstrates AMP expression is related to arboviral infection as well.

Previous studies revealed that the complement-like factor identified in arthropods shares common ancestry in vertebrates and invertebrates as an immune defense mechanism^31,39,40^. The complement system mediates virus elimination during an early phase of infection, and the viral recognition cascade composed of the complement-related protein is a crucial effector in opposing flaviviral infection through an axis regulating the expression of AMPs^17^. The identified and characterized antiviral host factors, particularly complement-like proteins, indicate that MCR limits DENV and YFV infection in *Ae. aegypti*^17^.

In insects, MCR belongs to the iTEP family, which exhibits high similarity to mammalian complement C3. The thioester domain of iTEPs binds the surface of microbes via a covalent bond and triggers the phagocytosis and opsonization of microbes. For instance, MCR of *D. melanogaster*, which shares multiple common domains with iTEPs, has been reported to target pathogenic yeast, *Candida albicans*, for phagocytosis^41^. *Anopheles* TEP1, which has been reported as a critical factor in pathogen elimination, eradicates *Plasmodium* spp.^30^ and recognizes parasites such as *Escherichia coli* and *Staphylococcus aureus* to target them for phagocytosis ^42^. A transgenic mosquito line with a TEP1 loss-of-function phenotype under a blood meal-inducible promoter exhibited increased viral protein and titers after an infectious blood meal, and the expression of transcription factor Rel2 and certain AMPs were inhibited^43^.

Mosquitoes have evolved mechanisms to tolerate persistent infection and developed efficient antiviral strategies to restrict viral replication to nonpathogenic levels in specific tissues^44^. AMPs are evolutionarily conserved components of important innate immune effectors that participate in responses to microbial infections in invertebrates^24^. The distinct activities against flavivirus in mosquitoes include AMP-mediated responses to viral infections in an early phase of invasion^23,33,34^. Furthermore, AMP genes may target and modulate flaviviruses at different time points^33^. Based on their positive charges, AMPs can electrostatically or hydrophobically associate with the components of the viral surface, subsequently resulting in flaviviral inactivation^19^. In mammals, AMPs have been demonstrated to efficiently eliminate bacteria and fungi^20^. In *Ae. albopictus*, our results revealed that five AMPs, including four CECs and one DEF, are upregulated by DTMUV infection. Among them, CECB and DEFC directly interacted with DTMUV. In the absence of the identified antiviral mechanisms, viral replication is higher than usual which may cause damage to the mosquito physiology and thereby decrease the mosquito life span.

In agreement with our finding, DEFs have a role in the antiviral activity, which is mediated directly through interactions with viral envelopes and indirectly through interactions with potential target cells to interfere with the cell-signaling pathways required for viral replication^45^. For instance, DEFC appeared to directly interact with the viral surface, which resulted in the flaviviral inactivation in *Ae. aegypti*^17^. In the case of CECs, CEC-like peptides limited DENV and chikungunya virus infection in *Ae. aegypti* salivary glands*,* indicating that CEC-like peptide is a key antiviral protector in the salivary glands^33,44^. Furthermore, the CEC–prophenoloxidase regulatory mechanism is a cross-species physiological function among mosquitoes including *Ae. albopictus, Ae. aegypti*, *Armigeres subalbatus*, and *Cx. quinquefasciatus*. For instance, CECB can regulate prophenoloxidase-3 (PPO3) expression in *Ae. albopictus* pupae via binding to TTGG(A/C)A motifs within PPO3 DNA that play crucial roles in cuticle formation during pupal development^46,47^.

The discovery of mosquito saliva proteins critical for pathogen transmission will enable us to better understand the mechanism through which arthropod-borne diseases establish early infection in the host and facilitate vector protein-based production. To illustrate, a prior study attempted to use human IgG antibody responses to Nterm-34 kDa peptide as a biomarker that reflects the intensity of human exposure to *Aedes* mosquito bites^28,48^, and it could be used as an immuno-epidemiological tool to estimate vector control in chikungunya and dengue transmission areas^49^. Furthermore, the anti-*Aedes* salivary extract antibody that reacts with proteins within the 34 kDa molecular weight range was used to assess the risk of vector exposure in the dengue-epidemic area^50^. Our further study may be generalizable to additional arbovirus and mosquito species. Theoretically, it is possible that administering specific antiviral agents to the host could modulate viral infectivity, perhaps in conjunction with traditional protein-based vaccines and therapeutics.

## Conclusion

To the best of our knowledge, this is the first study to elucidate the effect of 34 kDa in *Ae. albopictus*. This study proposed the possibility of deactivating anti-DTMUV processes in *Ae. albopictus* based on the following findings: (i) the 34 kDa salivary protein induced by a viral infection and (ii) the inducing of 34 kDa might cause interrupt the expression of AMPs, thereby facilitating virus replication and transmission. Further study would be required to discover the mysteries of 34 kDa salivary protein in terms of structures, mechanisms, and functions in the mosquito salivary gland.

## Methods

### Ethics statement and biosafety

The research plan for experiments involving mosquitoes in this study was reviewed and approved by the Chulalongkorn University Animal Care and Use Committee (Bangkok, Thailand; Animal Use Protocol No. 2131009 and 2231036), and it followed university regulations and policies governing the care and use of laboratory animals. All procedures were performed according to guidelines documented in the Ethical Principles and Guidelines for the Use of Animals for Scientific Purposes published by the National Research Council of Thailand. Moreover, this project was reviewed and approved by the Faculty of Veterinary Science Biosafety Committee of Chulalongkorn University (Biosafety Use Protocol No. IBC 2131004) and conducted according to their regulations and policies governing biosafety.

### Mosquitoes

*Ae. albopictus* mosquitoes (Bangkok strain), achieved from laboratory breeding for 29 generations, were kept at 28 °C under a 12 h/12 h light/dark cycle. Hatched larvae were transferred to plastic containers with sufficient water and fed grilled fish daily. Pupae were then collected and moved to a plastic container in a mosquito cage, and emerged mosquitoes were fed using cotton soaked with 10% sucrose solution. Three to five days post-eclosion (PE), female mosquitoes used for this study were immobilized by CO_2_ when retrieved from the cage.

### Bioinformatics

The sequences of 34 kDa genes in *Ae. albopictus* and *Ae. aegypti* were obtained from VectorBase (https://www.vectorbase.org/). The phylogenetic tree was constructed via the neighbor-joining method^51^ using MEGA 7 software based on the alignment of the sequences determined using Clustal W. The bootstrap consensus tree was inferred from 500 replicates. The sequence accession numbers of the 34 kDa genes incfluding *Ae. albopictus* 34 kDa (*AALF 004,424, AALF060952, AALF017483, AALF059151, AALF004421*) and *Ae. aegypti* 34 kDa (*AAEL003600, AAEL003601, AAEL003585*). *AAEL003600* was named *Ae. aegypti* salivary gland protein of 34 kDa or AaSG34 in the previous study^18^ and *AALF004421* was named 34 kDa throughout this study.

The sequences of iTEP genes in *Ae. albopictus, Ae. aegypti*, *An. gambiae*, and *D. melanogaster* were obtained from VectorBase (https://www.vectorbase.org/) and FlyBase (http://flybase.org). The phylogenetic tree was constructed via the neighbor-joining method^51^ using MEGA 7 software based on the alignment of the sequences determined using Clustal W. The bootstrap consensus tree was inferred from 500 replicates. The sequence accession numbers of iTEP genes followed the previous study^17,52^, including *Ae. albopictus* MCR (*AALF005612*), *Ae. aegypti* MCR (*AAEL012267*), *An. gambiae* MCR (*AGAP008407*), *An. gambiae* TEP3 (*AGAP010816*), *An. gambiae* TEP4 (*AGAP010812*), *An. gambiae* TEP6 (*AGAP010814*), *An. gambiae* TEP9 (*AGAP010830*), *An. gambiae* TEP10 (*AGAP010819*), *D. melanogaster* TEP1 (*CG18096*), *D. melanogaster* TEP2 (*CG7052*), *D. melanogaster* TEP5 (*CG13079*). *AAEL012267* was named as AeTEP1^31^ or AaMCR^17^ in the previous study, and *AALF004421* was named MCR throughout this study.

### Cell culture and virus

Baby hamster kidney-21 (BHK-21) cells were cultured in Modified Eagle’s Medium (Opti-MEM^®^ I, Gibco, USA) supplemented with 4% heat-inactivated fetal bovine serum, and 1% antibiotic–antimycotic (Cat. no. 15240062). The cells were maintained at 37 °C with 5% CO_2_. DTMUV cluster 2.1 strain DK/TH/CU-1, which constitutes a predominant cluster of DTMUV in Thailand, was kindly provided by the Department of Veterinary Microbiology, Faculty of Veterinary Science, Chulalongkorn University. As previously described, this virus was isolated from sick broiler ducks in Thailand’s disease outbreak area^53^. To produce the virus, DTMUV was propagated in 9-day-old embryonated duck eggs, harvested at 5 days post-infection, and stored at − 80 °C. To determine the viral titer, the Median tissue culture infectious dose (TCID_50_) was calculated. Mosquitoes were infected with DTMUV at approximately 1.0 × 10^7^ TCID_50_/mL.

### DTMUV infection

To avoid the possibility of bacteria-induced AMP expression in the mosquitoes for this study, dsLacZ- and ds34kDa- infected mosquitoes were the same colony that was achieved from laboratory breeding, not natural mosquitoes that were collected from the fields. Furthermore, to prepare the blood for this study, the sheep blood was washed to remove excess white blood cells, cytokines, and other allergen proteins from the supernatant, and the residue red blood cells were supplemented with 1% antibiotic–antimycotic (Cat. no. 15240062) to prevent bacterial and fungal contamination.

For oral infection, female mosquitoes were transferred into cylindrical cups with a nylon mesh top and starved via sucrose solution deprivation for 12 h. An infectious blood meal consisting of 1% antibiotic–antimycotic erythrocytes and DTMUV at a 1:1 ratio (1.25 × 10^6^ TCID_50_ in 500 µL) was wrapped in a stretched Parafilm-M membrane. The starved mosquitoes in the cups were allowed to consume the infectious blood meal for 30 min. The mosquitoes in the cups were then stored in the cage, where they were allowed to feed on a 10% sucrose solution.

For intrathoracic infection, female mosquitoes were immobilized by CO_2_ and intrathoracically inoculated with 69 nL of DTMUV (0.69 × 10^2^ TCID_50_ in 69 nL) using a Nanoject II Auto-Nanoliter Injector (Drummond Scientific, USA). After the injection, the mosquitoes were immediately transferred to cylindrical cups with a nylon mesh top. A cotton soak with 10% sucrose solution was placed onto the nylon mesh. The cup of injected mosquitoes was then stored in the cage.

### RNA extraction and reverse transcription

The samples were collected in 1.5-mL tubes containing 0.2 mL of 1 × PBS. The samples were stored at − 80 °C until further use. The samples were homogenized with a rotor–stator homogenizer at room temperature for 5 min and centrifuged at 3000× *g* and 4 °C for 5 min to produce a 10% suspension. Total RNA was extracted using a Viral Nucleic Acid Extraction Kit II (Geneaid, Taiwan). The RNA concentration was quantified using a spectrophotometer (NanoDrop 2000, Thermo Fisher Scientific, Waltham, MA, USA) and diluted in DEPC-H_2_O to a concentration of 1 μg/μL. The RNA samples were reverse-transcribed to cDNA with 250 ng of RNA/sample using the ImProm-II™ Reverse Transcription System (Promega, Madison, WI, USA) following the manufacturer’s protocol. The cDNA samples were then stored at − 20 °C until further use. Gene expression was analyzed by polymerase chain reaction (PCR) using ProTaq Plus DNA Polymerase (Protech). The ribosomal protein S7 gene was used as an internal control.

### Real-time quantitative PCR (RT-qPCR)

The RT-qPCR system used in this study consisted of TaqMan probes and the SYBR Green dye binding system. For the TaqMan probe system, real-time PCR using TaqMan™ Fast Advanced Master Mix (Applied Biosystems, USA) was performed in a MicroAmp Fast Optical 96-well reaction plate using the Step One Plus™ Real-Time PCR System (Applied Biosystems). Primers and TaqMan probes were specific for the envelope (E) gene of DTMUV. The primer set was 5′-TGTCTTATGCAGGTACCGATG-3′ (forward) and 5′-CGTATGGGTTGACTGTTATC A-3′ (reverse), and the TaqMan probe sequence was FAM-AGTTCCCATATCCATGTC-MGB^53^. The PCR program consisted of initial denaturation at 95 °C for 3 min followed by 40 cycles at 94 °C for 3 s and 60 °C for 40 s. Fluorescence readings were measured at 72 °C after each cycle, and the target gene signal was detected and analyzed using the Step One Plus™ Real-Time PCR System. The standard curve was obtained via serial dilutions of the recombinant plasmid (pUC-19) containing the E gene of DTMUV. The linear regression curve was obtained by plotting the mean C_t_ values with the copy number of each plasmid dilution. The absolute quantity of DTMUV from the samples was normalized per 250 ng of total RNA.

For the SYBR Green dye binding system, the cDNA sample was quantified using PowerUp™ SYBR™ Green Master Mix (Applied Biosystems) in a MicroAmp Fast Optical 96-well reaction plate. SYBR Green binds to the minor groove of DNA, and the target gene is quantified by detecting the resulting fluorescence signal. The qPCR primers were designed using ABI Primer Expression Software. The accession number and primers used for RT-qPCR in this study are listed in Table 1. The PCR program consisted of initial denaturation at 95 °C for 3 min followed by 40 cycles at 94 °C for 3 s and 60 °C for 40 s. Fluorescence readings were measured at 72 °C after each cycle, and the target gene signal was detected and analyzed using the Step One Plus™ Real-Time PCR System. The relative quantification results were normalized using the ribosomal protein S7 gene as an internal control.

**Table 1:** Primers and probe for RT-qPCR.

Primer name	Accession number/GenBank	Forward primer 5′-Sequence-3′	Reverse primer 5′-Sequence-3′	Probe
DTMUV Envelope Gene	MH460536.1	TGTCTTATGCAGGTACCGATG	CGTATGGGTTGACTGTTATCA	FAM-AGTTCCCATATCCATGTC -MGB
34 kDa salivary protein	AALF004421	TCAAAAGCGCCATCTACAGT	CTGGGTCTTCAATACCTCCA	
MCR	AALF005612	TCCGCCCCGGTCAGGTTTACAA	TCGACGCCATCCCGGGAGATAC	
Ribosomal Protein S7	AALF016123	GCGCGTCAAGCTGGATGGATCGCA	TCCGGTCAGCTTCTTGTACACCGA	
CECA	AALF000656	GCTGGGTCAAACCGAAGCCGG	ATTTTCCAAGTGCCTTGATTCC	
CECB	AALF014650	ATGAACTTCAACAAACTGTTCC	AACACACGCTTGCCGGCCTTT	
CECD	AALF043934	ACTTCAGCAAACTGTTTGCGCTTG	CCTTGAACACTCGCTTGCCGA	
CECE	AALF024715	GCTCTGCTTTTCACTGCCCAAACC	TGCAGCCCCAGAACAGTTGGGA	
CECG	AALF026733	CTGGCGGACGAGGTTCGTTCTTT	ACCCCGAATCCACTCAGCAGATCG	
CECI	AALF012131	AAGAAGCTGTTCATCTTCGTC	TCACTTTGTCGACAGCATGGGC	
DEFA	AALF065006	AACTTGCGCCTCAAGCGGGCCACC	TCAGTTCCGGCAGACGCACACCTT	
DEFC	AALF008821	GAGCTGTCCGACGATGTCCGCTC	GCACTATCCCCAACACCGAA	
DEFD	AALF003252	CTCGTTCAAGCGATATCAGTTC	TCGTCCGCCAGCACCGATTC	
DEFE	AALF008822	GAATTGCTACGCCGAAAGCGC	ACAAACGCAGACTCCCCGATC	
ATT	AALF048664	GAATCCCTAGCTCAGTTTCA	AAACTGGTCCTCCCTTGGTGTT	
DPT	AALF044217	TTCGGGGCTACGCACCAGCAGGA	CGTCGCCCTGAAATCCACCGAA	
GAM	AALF001757	CGAGAGCCAAAACCTGTGCC	TGCAGCTGGCTATCGAATTCGC	

### Double-stranded RNA (dsRNA) synthesis

Primer design for dsRNA synthesis should be approximately 15–20 bp gene-specific sequence with the T7 promoter (5′-TAATACGACTCACTATAGGG-3′) that was incorporated into all forward and reverse primers^54^. The accession number and primers used for dsRNA synthesis were listed in Table 2. The target gene fragments with the T7 polymerase promoter were amplified using Platinum^®^ Taq DNA Polymerase (Invitrogen, Waltham, MA, USA). Fragments were amplified and cloned with a pCR2.1 TOPO vector (Invitrogen) at 23 °C for 30 min. The constructed plasmid was transformed into *E. coli* HIT-DH5α competent cells. Plasmids from positive colonies were purified using a FavorPrep™ Plasmid DNA Extraction Mini Kit (Favorgen, Taiwan) and sequenced to confirm that the cDNA was generated in-frame. A restriction enzyme was used to digest the plasmids, and the fragments were separated by 1% agarose gel electrophoresis, excised from the gel, and purified using a FavorPrep™ Gel/PCR Purification Kit (Favorgen). The purified PCR product was used as the template for synthesizing the dsRNA. Sense and antisense strands of RNA are synthesized and annealed together in an in vitro transcription process^54^ using a T7-Scribe™ Transcription Kit (Epicenter). The reaction was performed at 37 °C for 4–12 h. Ammonium acetate (stop solution) was added to the dsRNA to stop the reaction, and the dsRNA was purified and dissolved in DEPC-H_2_O. All kit systems were used according to the manufacturer’s recommendation. The dsRNA synthesis in the control group was specific to the LacZ operon of *E. coli*-*Thermococcus kodakarensis* shuttle vector pTNTrpE^55^. The dsRNA was diluted to a final concentration of 5 μg/μL. At 3–5 days PE, female mosquitoes were injected with 207 nL of dsRNA (5 μg/μL) using a Nanoject II Auto-Nanoliter Injector. Silencing efficiency was confirmed by collecting the total RNA of the mosquito salivary glands for RT-qPCR.

**Table 2:** Primers for dsRNA synthesis.

Primer name	Accession number/GenBank	Forward primer 5′-Sequence-3′	Reverse primer 5′-Sequence-3′
T7-34 kDa	AALF004421	TAATACGACTCACTATAGGGCGAAGAGAAGCTGAACGACC	TAATACGACTCACTATAGGGCACAGCACCAACCGATCC
T7-LacZ	MG920814.1	TAATACGACTCACTATAGGGTTTCCCCGTCAAGCTCTAAA	TAATACGACTCACTATAGGGAATCATGCGAAACGATCCTC
T7-CECA	AALF000656	TAATACGACTCACTATAGGGATGAACTTCAACAAG	TAATACGACTCACTATAGGGTCATTTTCCAAGTGC
T7-CECB	AALF014650	TAATACGACTCACTATAGGGATGGATATTATCT TTC	TAATACGACTCACTATAGGGTTATCTTCCCAGGGC
T7-CECE	AALF024715	TAATACGACTCACTATAGGGATGAACTTCAAGTGT	TAATACGACTCACTATAGGGTTCACGGATTTTCTG
T7-CECI	AALF012131	TAATACGACTCACTATAGGGCTGTTCATCTTCGTC	TAATACGACTCACTATAGGGCGTTTTCACGGATTT
T7-DEFC	AALF008821	TAATACGACTCACTATAGGGATGCGTACCCTCACC	TAATACGACTCACTATAGGGCCGCTTGGTAAGATT

### Serine protease inhibition

The serine protease inhibitor (SPI), Pefabloc^®^ SC AEBSF, 4(2-Aminoethyl)-benzenesulfonyl fluoride hydrochloride (Sigma-aldrich), was used at a final concentration of 1 mg/mL in PBS^32^. PBS was used as the control. At 3–5 days PE, female mosquitoes were injected with 69 nL of the inhibitor using a Nanoject II Auto-Nanoliter Injector. The silencing efficiency was confirmed by collecting the total protein of the mosquito salivary glands for Western blot analysis.

### Protein extraction and Western blot analysis

Mosquito salivary gland proteins were extracted by placing salivary glands in 100 µL of protein lysis buffer as previously described^56^. The homogenized suspensions were centrifuged at 15,700× *g* for 30 min. The supernatant was transferred to a QIA shredder™ column (Qiagen) and centrifuged at 15,700× *g* for 30 min. The eluted samples were collected and transferred to new tubes. Proteins extracted from the salivary gland were fractionated by protein electrophoresis on a 12% polyacrylamide gel and transferred to a 0.45-µm polyvinylidene fluoride membrane (Millipore). The membranes were then blocked with 5% skim milk in 1 × PBS containing 0.2% Tween-20 (PBST) for 30 min at room temperature. The membranes were incubated with the primary antibody against serine protease (Cat no. NBP1-79,488) at a 1:6000 dilution in 0.2% PBST at 4 °C overnight. The membranes were washed and then incubated with the secondary antibody (horseradish peroxidase (HRP)-conjugated polyclonal IgG) a 1:10,000 dilution in 0.2% PBST for 1 h at 4 °C. The membrane was washed in 0.2% PBST and developed in WesternBright™ ECL (Advansta Inc.) as a substrate for HRP. The bands were captured using the ChemiDoc™ Imaging System (Bio-Rad). The intensity of bands with positive signals was quantified using ImageJ software (National Institutes of Health, Bethesda, MD, USA).

#### Median tissue culture infectious dose (TCID_50_) assay

The control and experimental mosquitoes were inoculated with DTMUV via intrathoracic injection. DTMUV-infected saliva was collected by an artificial meal feeder as described previously ^58^ at 7 days post-infection. Briefly, a feeding solution containing 1 × PBS and 1 mM adenosine triphosphate at a final concentration of 10 μM was prepared. The feeding solution was wrapped in a stretched Parafilm-M^®^ membrane and placed on top of a container covered with nylon mesh, in which 200 infected mosquitoes were allowed to feed on the meal. The feeding solution containing infectious saliva was transferred to a microtube and centrifuged in 0.22-μm centrifuge tube filters at 12,000× *g* for 30 min at 4 °C. The filtered saliva samples were adjusted to an equivalent protein concentration, and serial tenfold dilutions were prepared using Opti-MEM medium. Confluent BHK-21 cell monolayers in 96-well plates (Corning, Corning, NY, USA) were washed with 1 × PBS and inoculated with the dilutions (100 µL/well) at 37 °C and 5% CO_2_. After 5 days of incubation, the inoculate were removed, and the cells were fixed with 100 µL of 3.7% formaldehyde for 30 min at room temperature. After the fixation, the residual formaldehyde was washed out using 0.5% PBST. Immunostaining for the cells was performed with primary antibody (50 µL/well) against flavivirus (monoclonal mouse IgG anti-flavivirus, GeneTex, GTX127277) containing 1% bovine serum albumin (BSA) in 0.5% PBST (dilution, 1:400) for 2 h at room temperature. After the primary antibody incubation, the residual antibody was washed out with 0.5% PBST. The cells were then stained with 50 µL/well secondary antibody (HRP-conjugated polyclonal IgG) containing 1% BSA in 0.5% PBST (dilution, 1:300) for 1 h at room temperature. After the secondary antibody incubation, removed and washed out the residual with 0.5% PBST. The cells were developed in the presence of AEC substrate (50 µL/well, Advansta Inc.) as a substrate for HRP for 30 min at room temperature. The cells were washed with tap water and dried for 1 h at room temperature. TCID_50_ was calculated as TCID_50_/mL to quantify virus infectivity.

### Statistical analysis

The data were analyzed using an unpaired* t*-test for all independent experiments. All statistical analyses were performed using GraphPad Prism 9.3.1 software (GraphPad Software Inc., San Diego, CA, USA) and *p* < 0.05 indicated statistical significance (Supplemenatry Information).

## Supplementary Information

Supplementary Information 1.

Supplementary Information 2.

Supplementary Information 3.

## Data Availability

All data generated or analyzed during this study are included in this published article and its supplementary information files.
