# Supplementary figures and images for: 34-kDa salivary protein enhances duck Tembusu virus infectivity in the salivary glands of Aedes albopictus by modulating the innate immune response
Source: Sci Rep. 2023 Jun 5;13:9098. doi: 10.1038/s41598-023-35914-x (PMC10241908; doi:10.1038/s41598-023-35914-x)

## Work flow for qPCR analysis

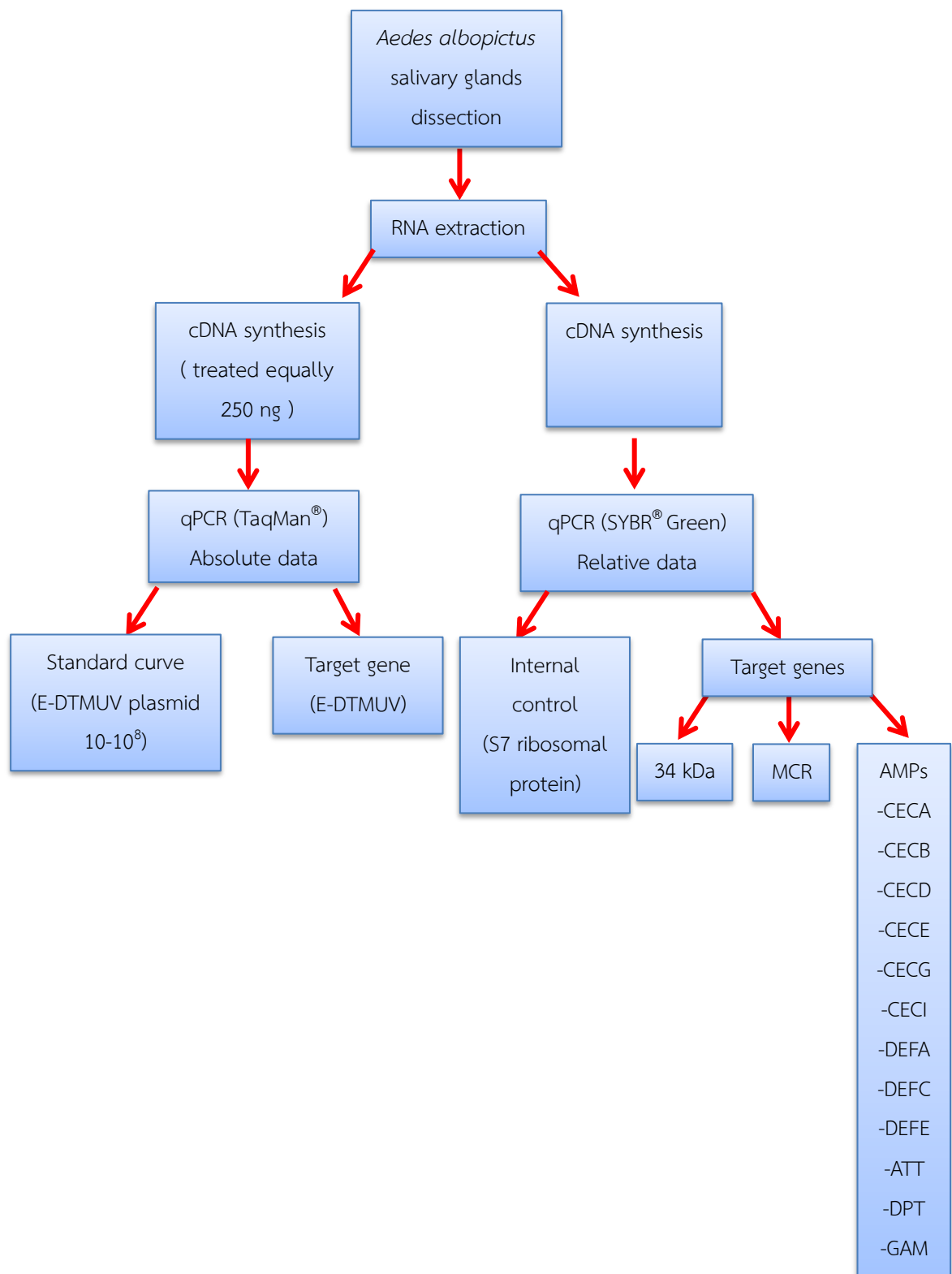

Supplement: Supplementary file 1 — Supplementary Information 1. [file 41598_2023_35914_MOESM1_ESM.pdf]
