# Supplementary material for: 34-kDa salivary protein enhances duck Tembusu virus infectivity in the salivary glands of Aedes albopictus by modulating the innate immune response
Source: Sci Rep. 2023 Jun 5;13:9098. doi: 10.1038/s41598-023-35914-x (PMC10241908; doi:10.1038/s41598-023-35914-x)
Supplement: Supplementary file 2 — Supplementary Information 2. [file 41598_2023_35914_MOESM2_ESM.pdf]

Supplementary legends

Supplementary figure_Workflow for qPCR analysis

Supplementary data_Raw data
