# Supplementary material for: 34-kDa salivary protein enhances duck Tembusu virus infectivity in the salivary glands of Aedes albopictus by modulating the innate immune response
Source: Sci Rep. 2023 Jun 5;13:9098. doi: 10.1038/s41598-023-35914-x (PMC10241908; doi:10.1038/s41598-023-35914-x)
Supplement: Supplementary file 3 — Supplementary Information 3. [file 41598_2023_35914_MOESM3_ESM.pdf]

- Detect S7 ribosomal protein for Fig.3-5 and other experiment samples

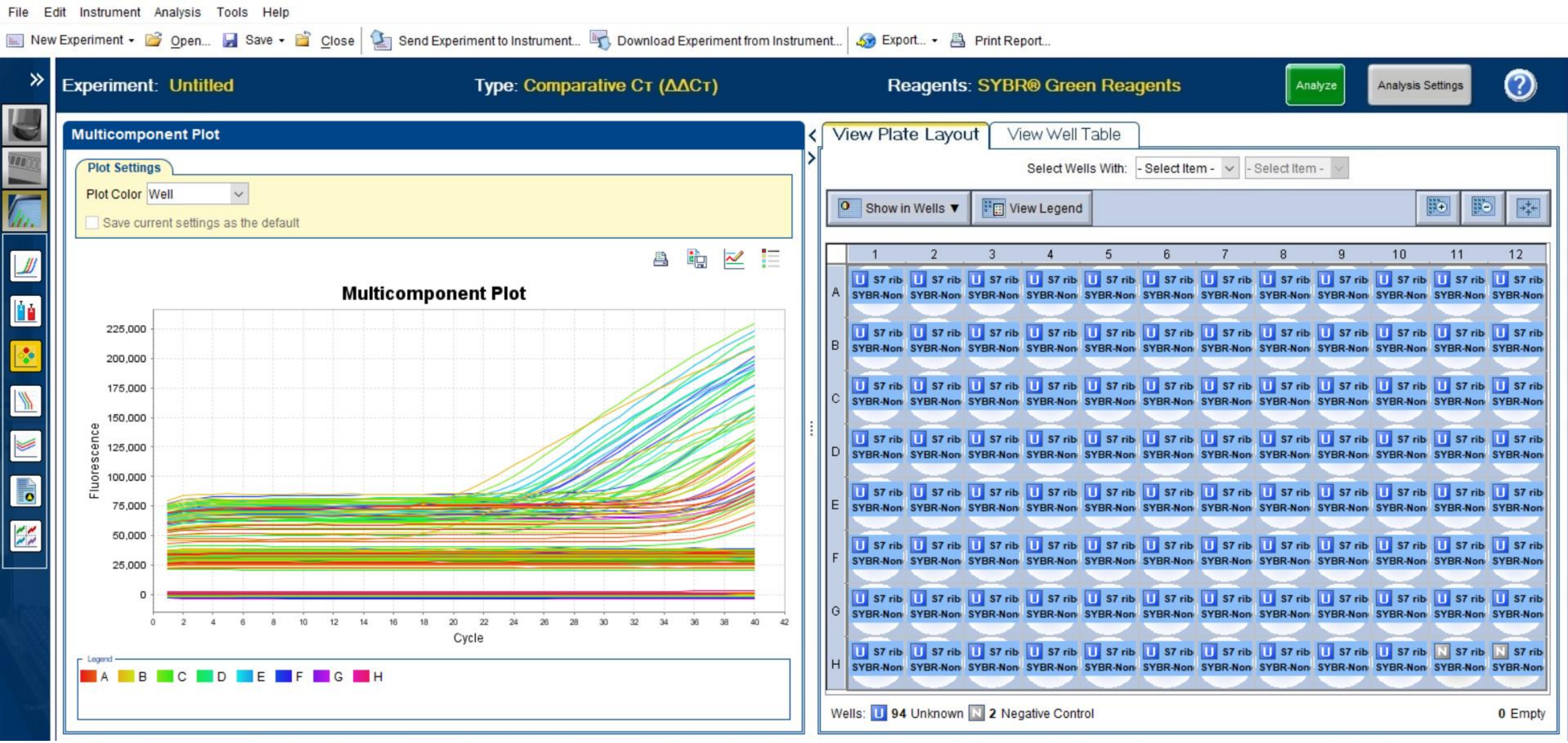

- Detect 34 kDa for Fig.3(B)-4(A) and other experiment samples

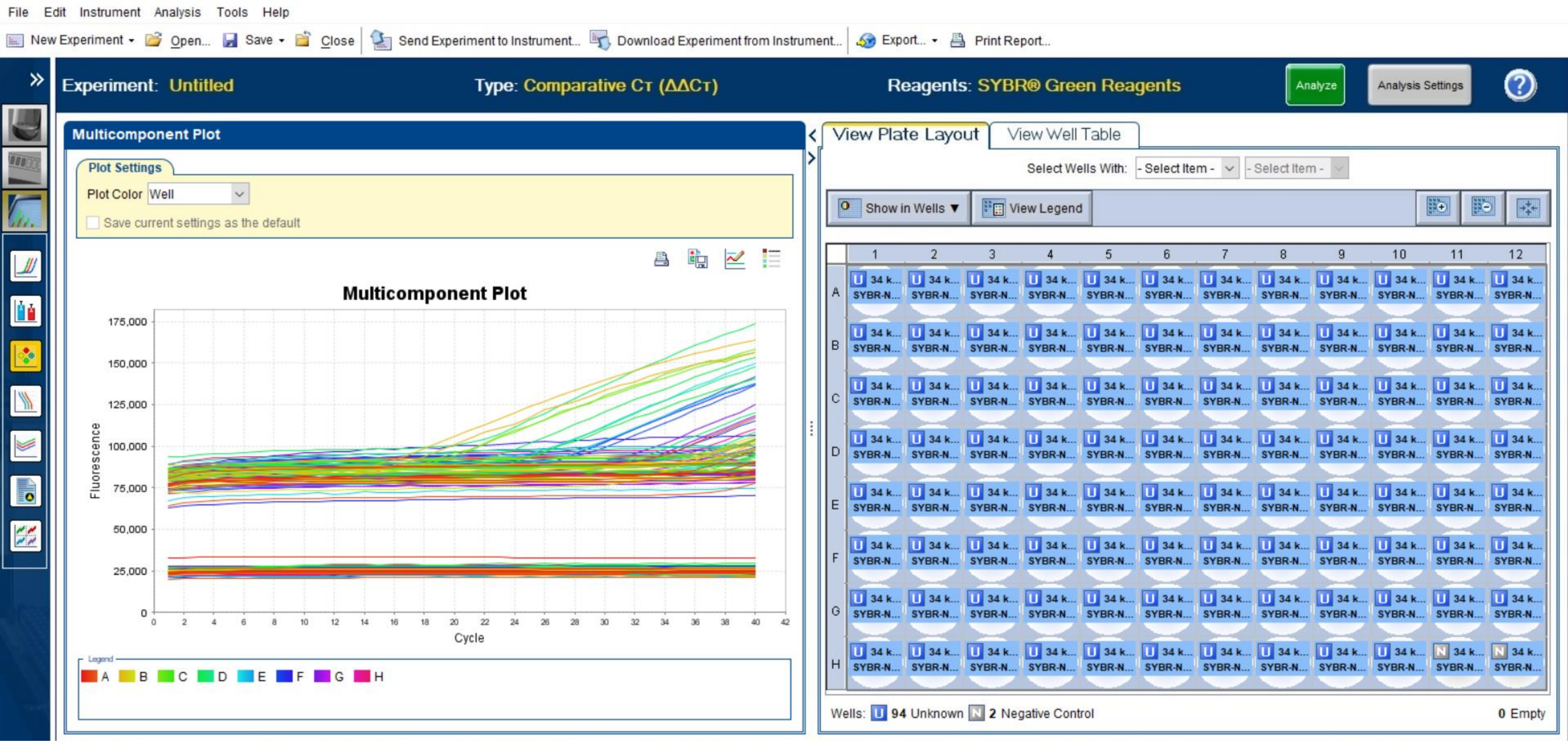

- Detect MCR for Fig.3(C)-4(B)-5(C) and other experiment samples

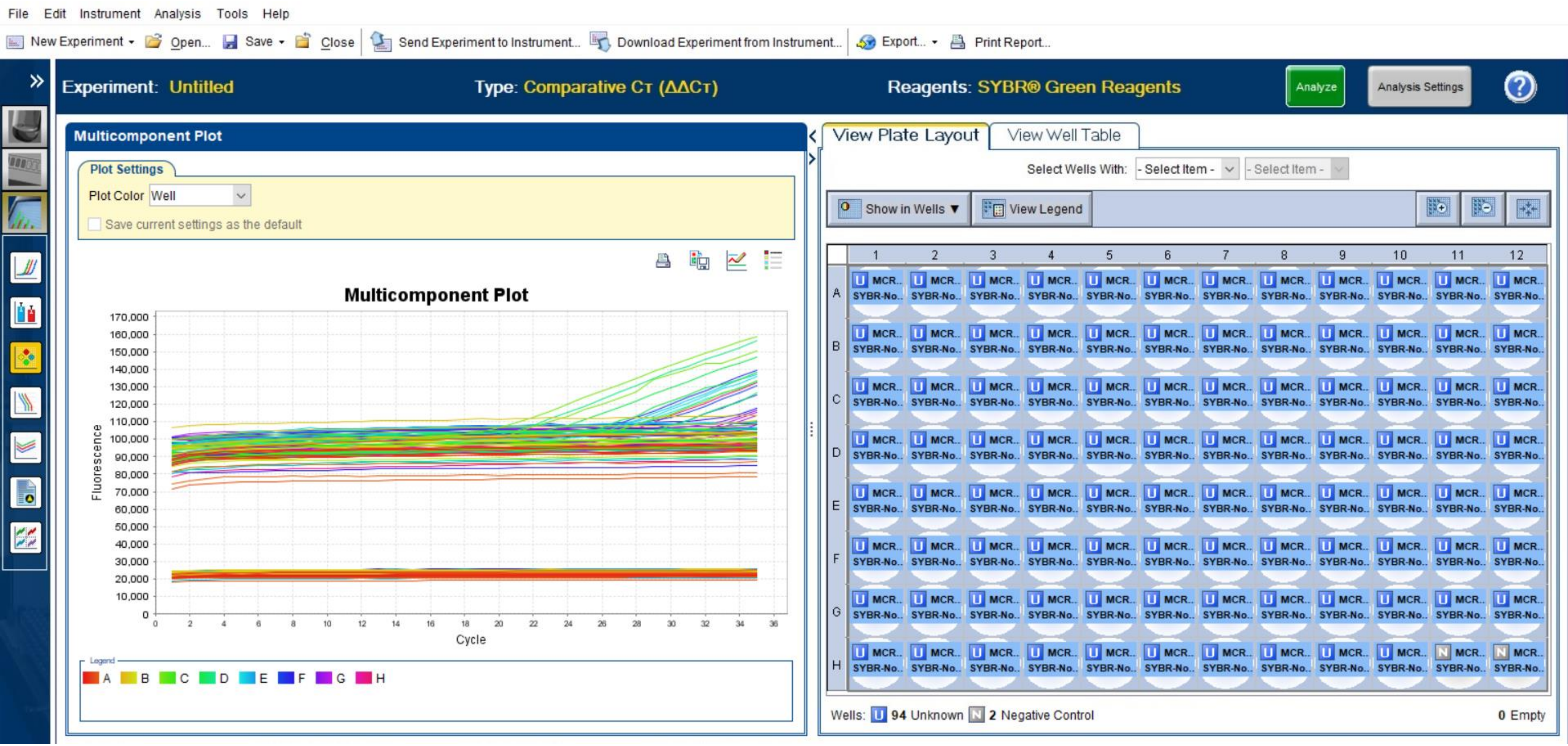

- Detect AMPs (CECA, CECB, CECD, CECG, CECI) for Fig.4(C) and other experiment samples

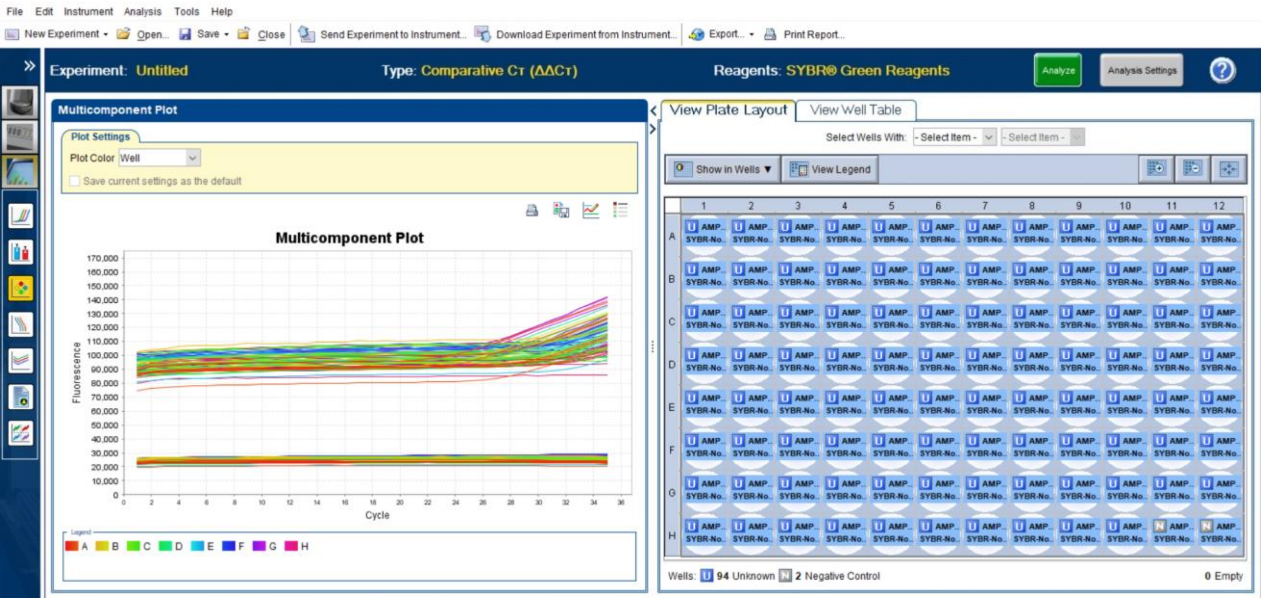

- Detect AMPs (DEFA, DEFC, DEFD, DEFE, ATT, DPT, GAM) for Fig.4(C) and other experiment samples

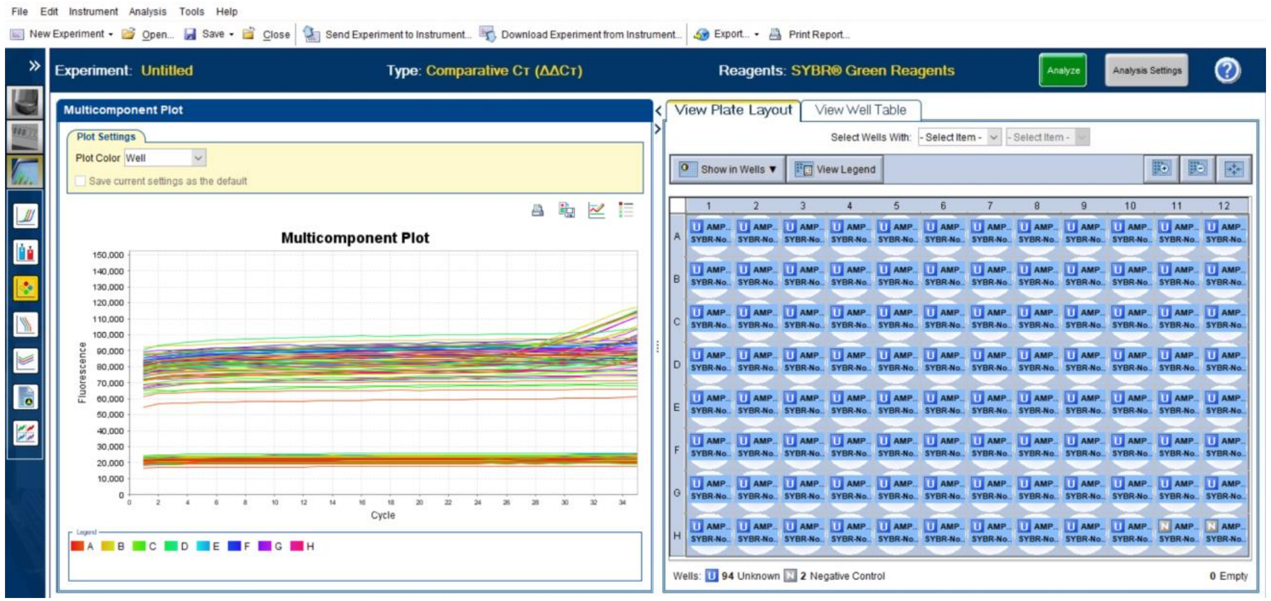

- Detect AMPs (CECA, CECB, CECD, CECG, CECI) for Fig.5(D) and other experiment samples

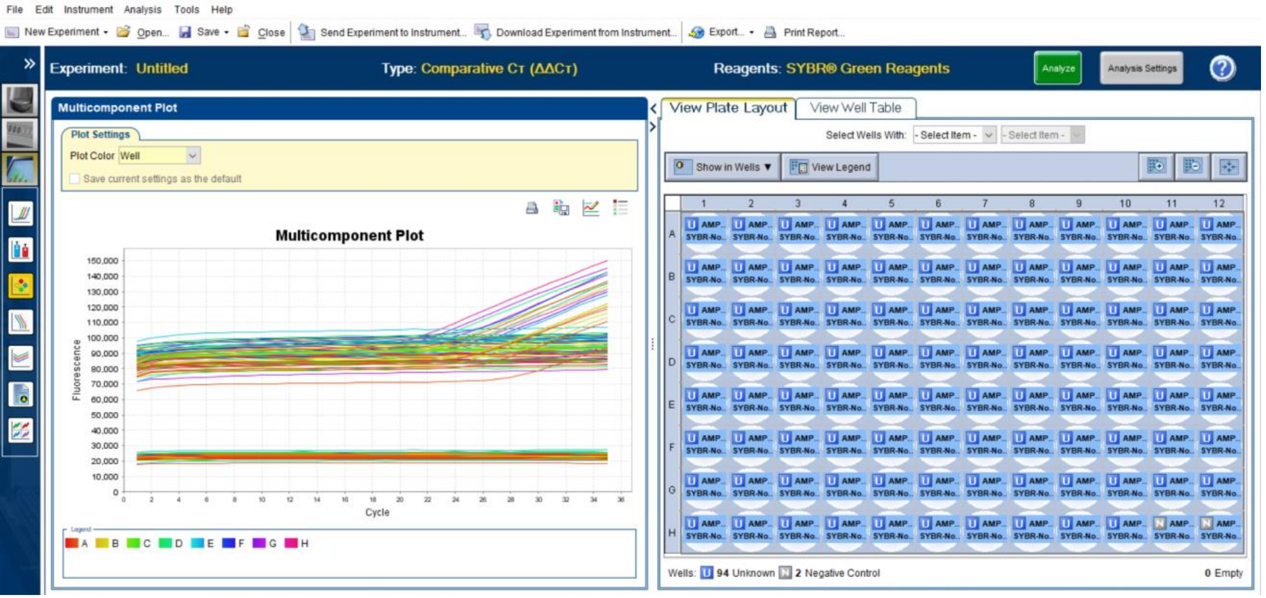

- Detect AMPs (DEFA, DEFC, DEFD, DEFE, ATT, DPT, GAM) for Fig.5(D) and other experiment samples

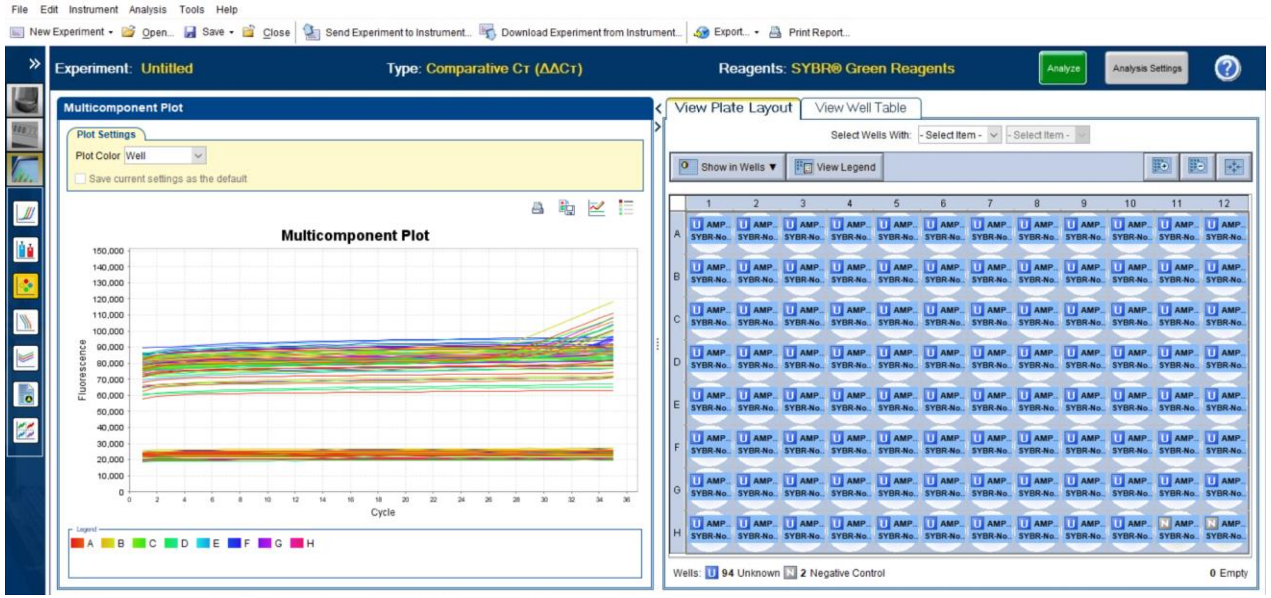

- Detect DTMUV for Fig.3(A)-4(D)-5(E) and other experiment samples

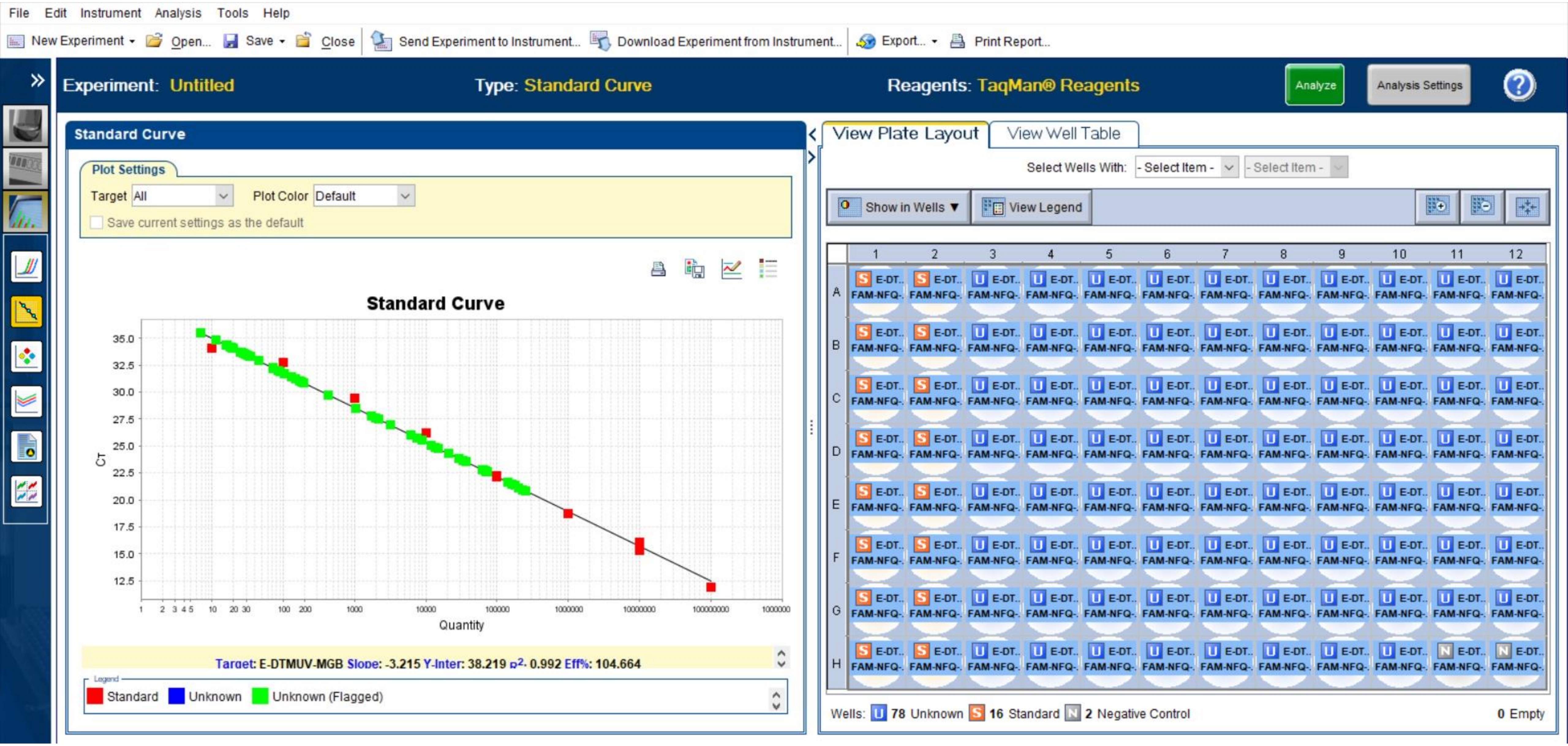

- Detect DTMUV for Fig.6 (LacZ dsRNA and 34 kDa dsRNA samples)

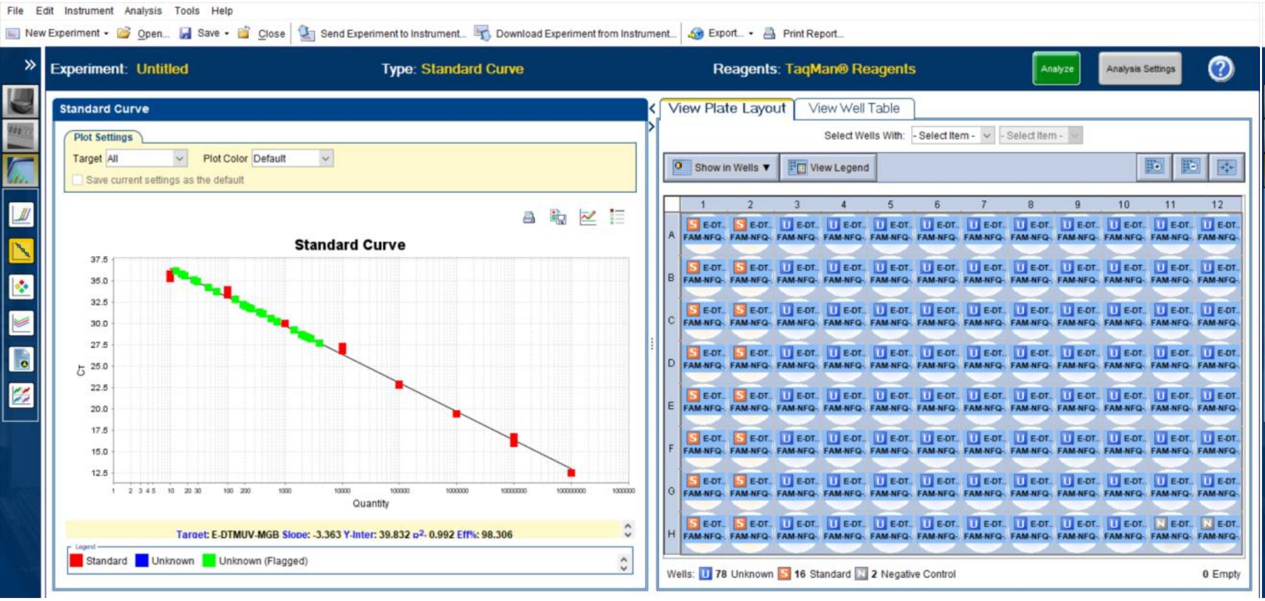

- Detect DTMUV for Fig.6 (34 kDa+CECA dsRNA and 34 kDa+CECB dsRNA samples)

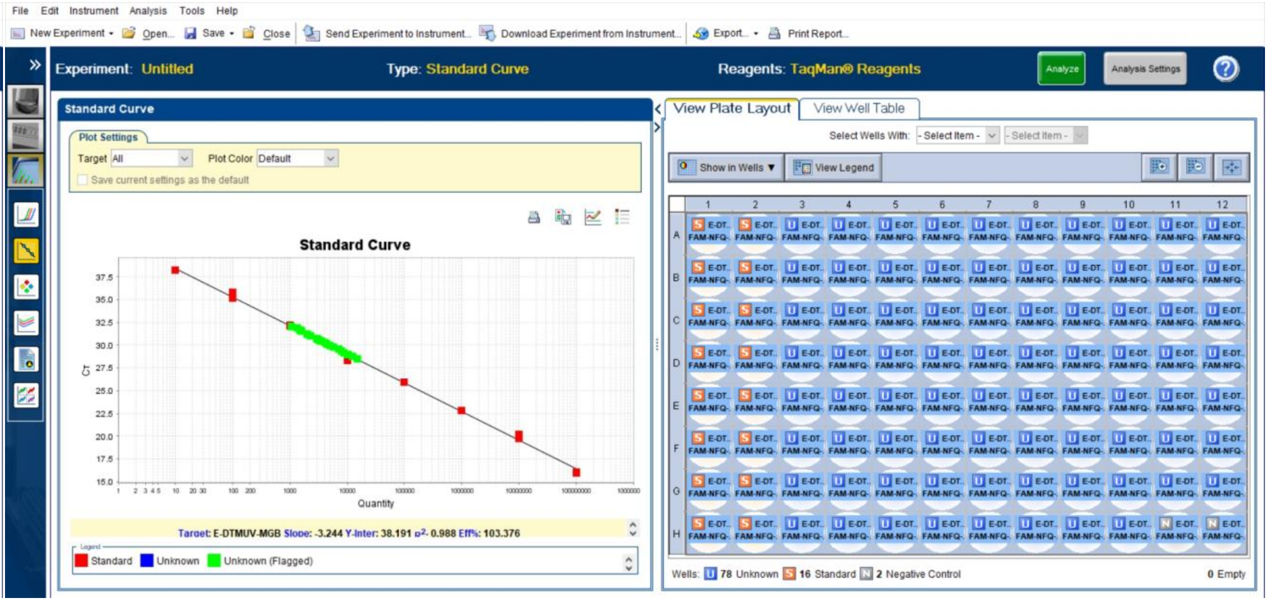

- Detect DTMUV for Fig.6 (34 kDa+CECE dsRNA and 34 kDa+CECI dsRNA samples)

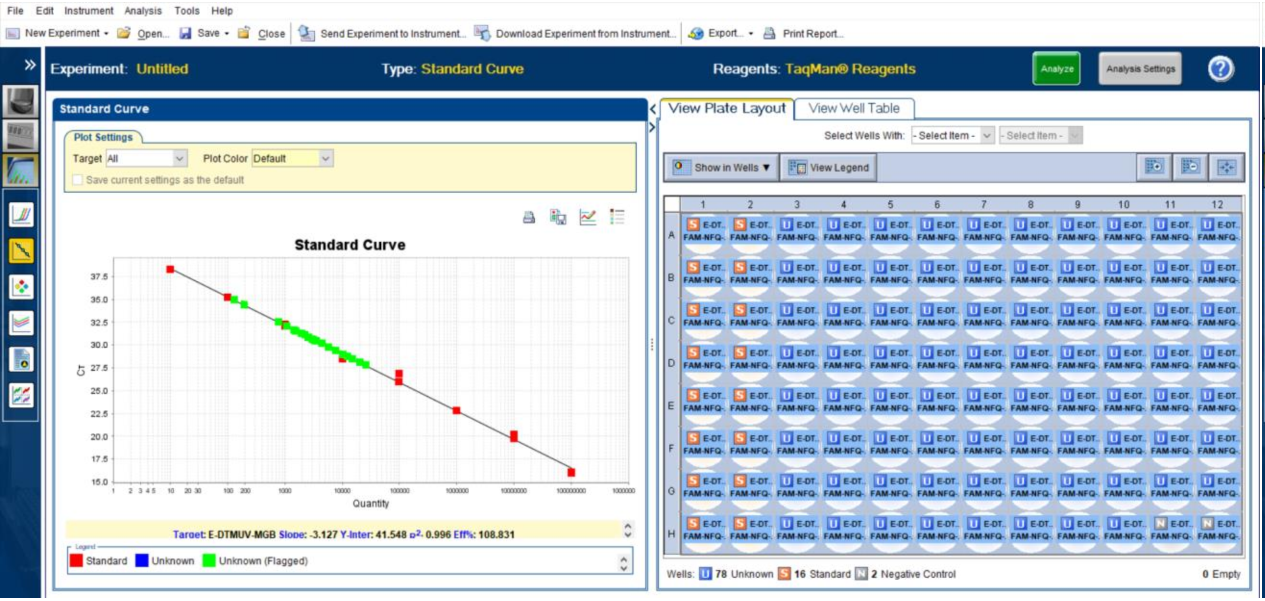

- Detect DTMUV for Fig.6 (34 kDa+DEFC dsRNA sample and other experiment samples)

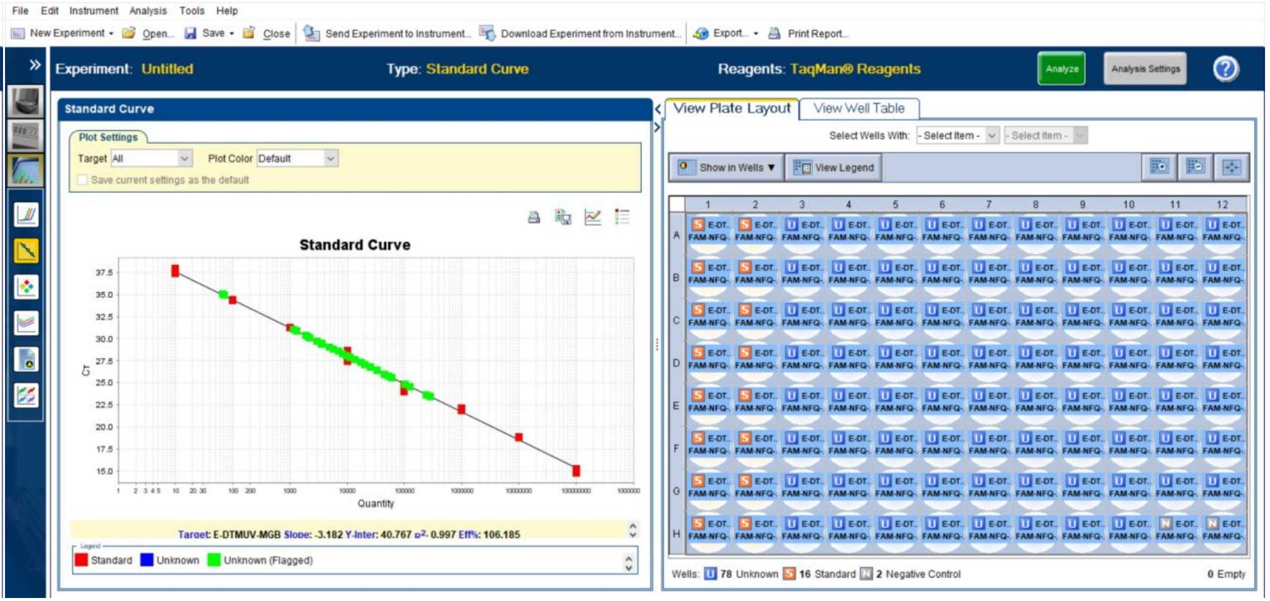

- Detect serine protease 34 kDa for Fig.5(A)

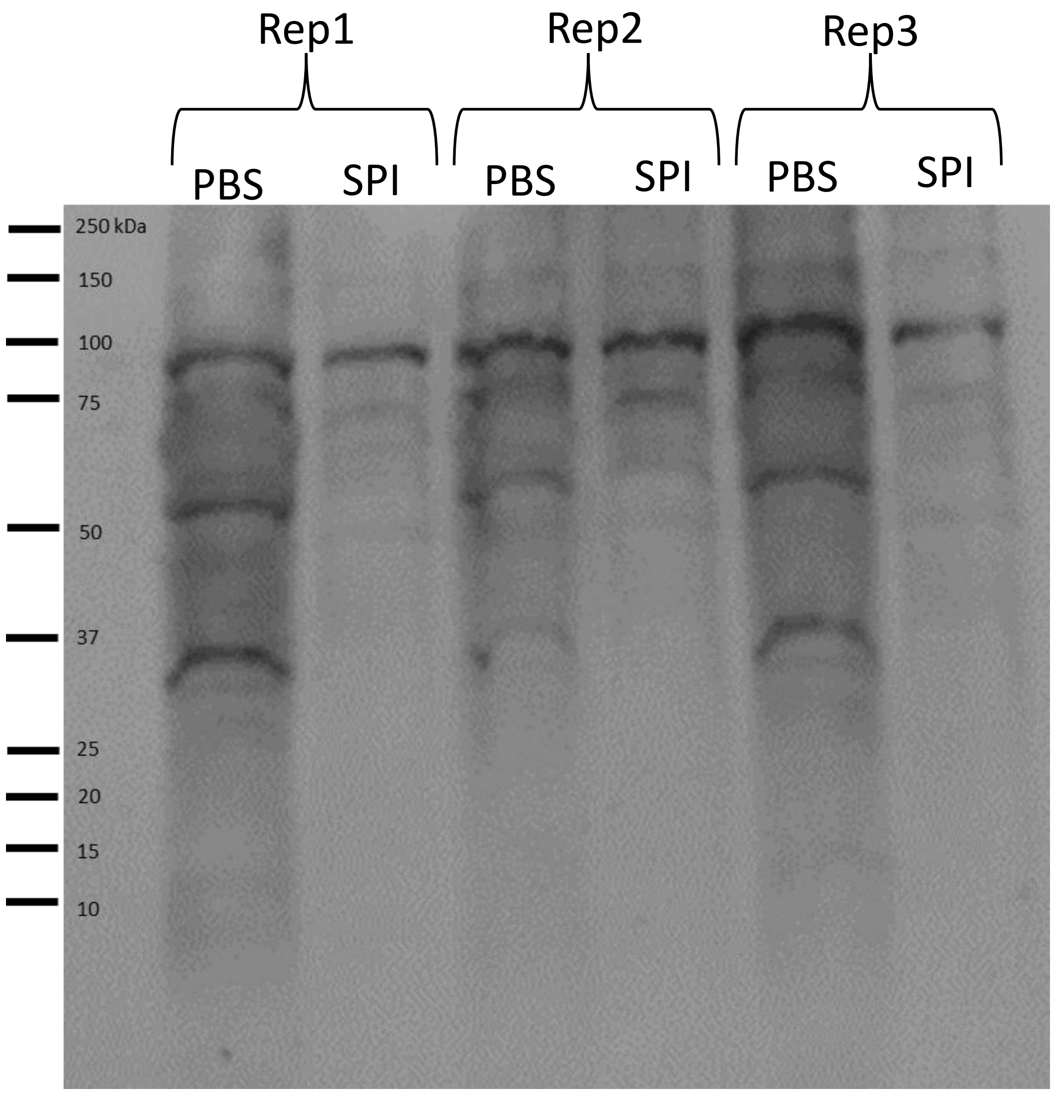

Original image

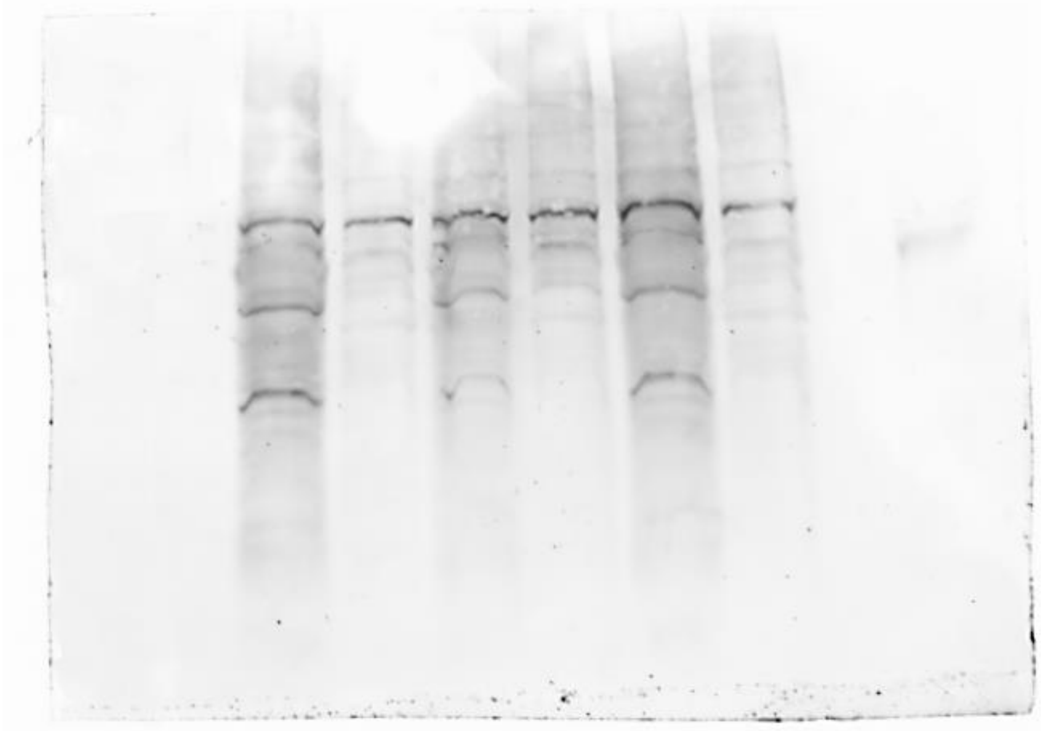

- Detect β-actin for Fig.5(A)

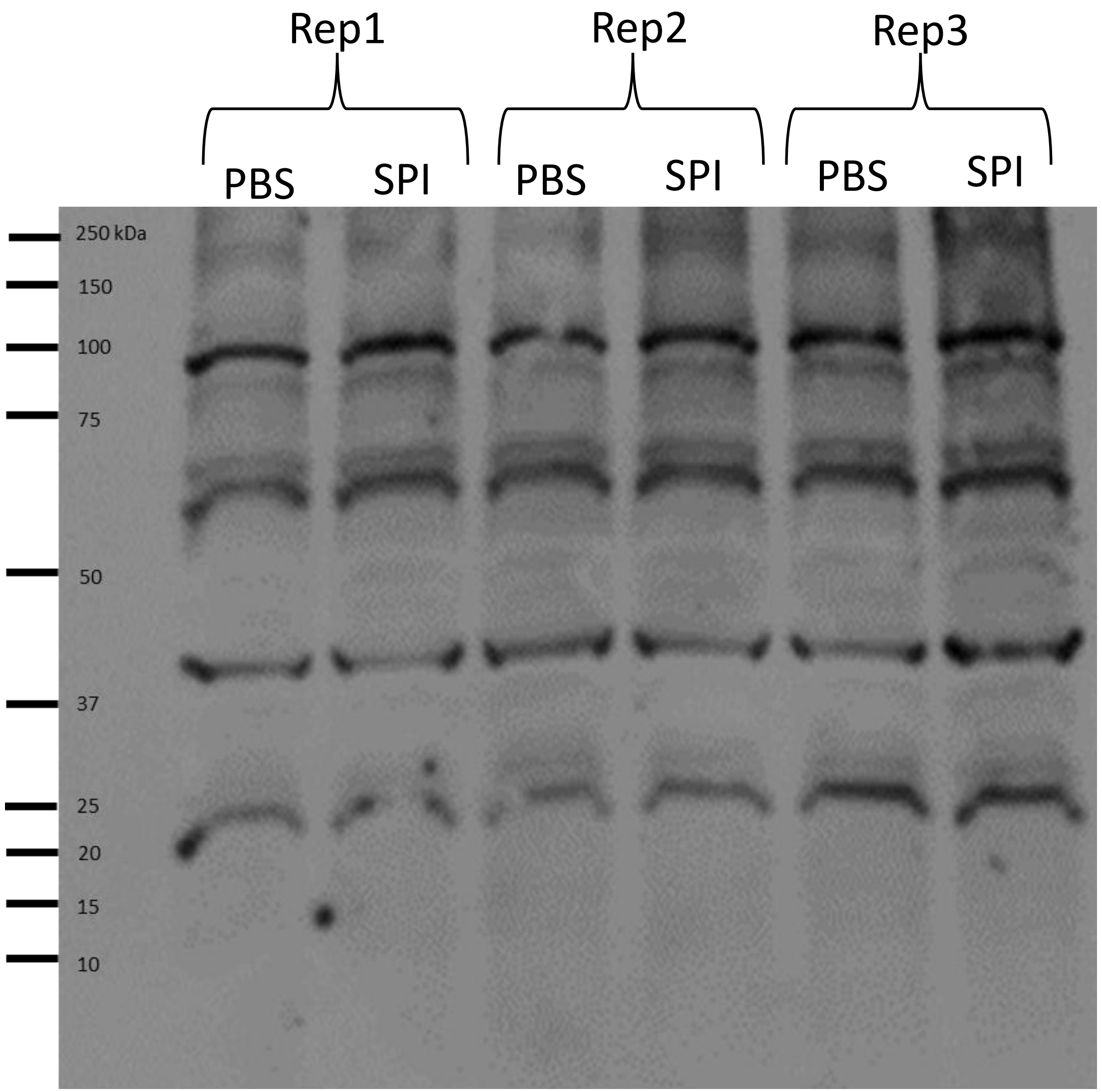

Original image

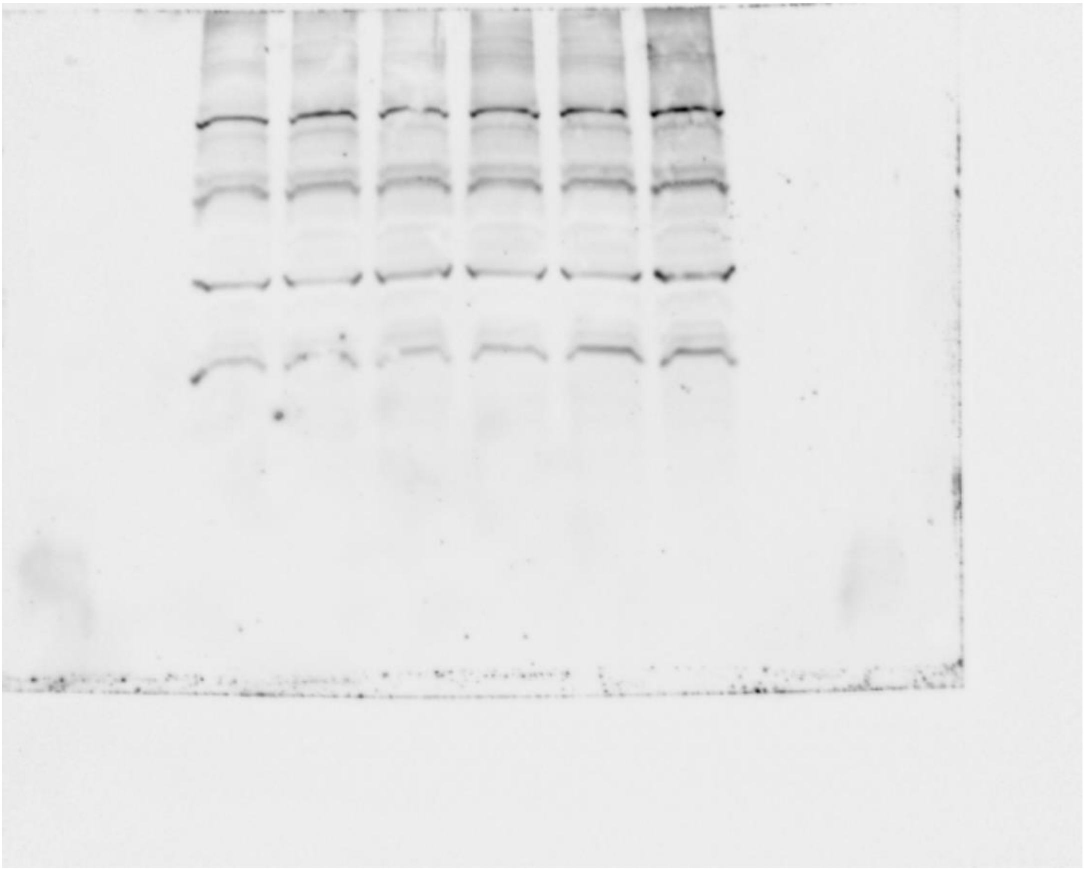

- TCID$_{50}$ for Fig.4(E)

Figure4 (dsRNA)

ds LacZ rep1	Dilution	bserved CPE and I		Cumulative value		Total	% Positive	PD	LogID50	D50/volu	TCID50/ml
		#Positive	#Negative	↑ Positiv	↓ Negativ						
	5.00E-01	4	0	17	0	17	100.00				
	1.00E-01	4	0	13	0	13	100.00				
	1.00E-02	4	0	9	0	9	100.00				
	1.00E-03	1	3	5	3	8	62.50	0.55556	-3.5556	3.59E+03	3.59E+04
	1.00E-04	1	3	4	6	10	40.00				
	1.00E-05	1	3	3	9	12	25.00				
	1.00E-06	1	3	2	12	14	14.29				
	1.00E-07	1	3	1	15	16	6.25				
	1.00E-08	0	4	0	19	19	0.00				

ds LacZ rep2	Dilution	bserved CPE and I		Cumulative value		Total	% Positive	PD	LogID50	D50/volu	TCID50/ml
		#Positive	#Negative	↑ Positiv	↓ Negativ						
	5.00E-01	4	0	16	0	16	100.00				
	1.00E-01	4	0	12	0	12	100.00				
	1.00E-02	4	0	8	0	8	100.00				
	1.00E-03	3	1	4	1	5	80.00	0.5	-3.5	3.16E+03	3.16E+04
	1.00E-04	1	3	1	4	5	20.00				
	1.00E-05	0	4	0	8	8	0.00				
	1.00E-06	0	4	0	12	12	0.00				
	1.00E-07	0	4	0	16	16	0.00				
	1.00E-08	0	4	0	20	20	0.00				

ds LacZ rep3	Dilution	bserved CPE and I		Cumulative value		Total	% Positive	PD	LogID50	D50/volu	TCID50/ml
		#Positive	#Negative	↑ Positiv	↓ Negativ						
	5.00E-01	4	0	16	0	16	100.00				
	1.00E-01	4	0	12	0	12	100.00				
	1.00E-02	3	1	8	1	9	88.89				
	1.00E-03	3	1	5	2	7	71.43	0.5625	-3.5625	3.65E+03	3.65E+04
	1.00E-04	2	2	2	4	6	33.33				
	1.00E-05	0	4	0	8	8	0.00				
	1.00E-06	0	4	0	12	12	0.00				
	1.00E-07	0	4	0	16	16	0.00				
	1.00E-08	0	4	0	20	20	0.00				

This is for ten-fold dilution.
This TCID50 is the proportion with virus volume in the experiment.

Innoculated virus(volume)/we 0.1 ml

Direction: Fill in the cells which are light blue.

ds 34 kDa rep1	Dilution	bserved CPE and I		Cumulative value		Total	% Positive	PD	LogID50	D50/volu	TCID50/ml
		#Positive	#Negative	↑ Positiv	↓ Negativ						
	5.00E-01	0	4	0	4	4	0.00				
	1.00E-01	0	4	0	8	8	0.00				
	1.00E-02	0	4	0	12	12	0.00				
	1.00E-03	0	4	0	16	16	0.00				
	1.00E-04	0	4	0	20	20	0.00				
	1.00E-05	0	4	0	24	24	0.00				
	1.00E-06	0	4	0	28	28	0.00				
	1.00E-07	0	4	0	32	32	0.00				
	1.00E-08	0	4	0	36	36	0.00				

ds 34 kDa rep2	Dilution	bserved CPE and I		Cumulative value		Total	% Positive	PD	LogID50	D50/volu	TCID50/ml
		#Positive	#Negative	↑ Positiv	↓ Negativ						
	5.00E-01	0	4	0	4	4	0.00				
	1.00E-01	0	4	0	8	8	0.00				
	1.00E-02	0	4	0	12	12	0.00				
	1.00E-03	0	4	0	16	16	0.00				
	1.00E-04	0	4	0	20	20	0.00				
	1.00E-05	0	4	0	24	24	0.00				
	1.00E-06	0	4	0	28	28	0.00				
	1.00E-07	0	4	0	32	32	0.00				
	1.00E-08	0	4	0	36	36	0.00				

ds 34 kDa rep3	Dilution	bserved CPE and I		Cumulative value		Total	% Positive	PD	LogID50	D50/volu	TCID50/ml
		#Positive	#Negative	↑ Positiv	↓ Negativ						
	5.00E-01	0	4	0	4	4	0.00				
	1.00E-01	0	4	0	8	8	0.00				
	1.00E-02	0	4	0	12	12	0.00				
	1.00E-03	0	4	0	16	16	0.00				
	1.00E-04	0	4	0	20	20	0.00				
	1.00E-05	0	4	0	24	24	0.00				
	1.00E-06	0	4	0	28	28	0.00				
	1.00E-07	0	4	0	32	32	0.00				
	1.00E-08	0	4	0	36	36	0.00				

This is for ten-fold dilution.
This TCID50 is the proportion with virus volume in the experiment.

Innoculated virus(volume)/we 0.1 ml

Direction: Fill in the cells which are light blue.

- TCID$_{50}$ for Fig.5(F)

Figure5 (Serine protease inhibitor)

PBS rep1

Dilution	pserved CPE and I(		Cumulative value		Total	6 Positive	PD	LogID50	D50/volu	TCID50/ml
	#Positive	#Negative	↑ Positiv	↓ Negativ						
5.00E-01	4	0	16	0	16	100.00				
1.00E-01	4	0	12	0	12	100.00				
1.00E-02	4	0	8	0	8	100.00				
1.00E-03	2	2	4	2	6	66.67	0.5	-3.5	3.16E+03	3.16E+04
1.00E-04	2	2	2	4	6	33.33				
1.00E-05	0	4	0	8	8	0.00				
1.00E-06	0	4	0	12	12	0.00				
1.00E-07	0	4	0	16	16	0.00				
1.00E-08	0	4	0	20	20	0.00				

PBS rep2

Dilution	pserved CPE and I(		Cumulative value		Total	6 Positive	PD	LogID50	D50/volu	TCID50/ml
	#Positive	#Negative	↑ Positiv	↓ Negativ						
5.00E-01	4	0	17	0	17	100.00				
1.00E-01	4	0	13	0	13	100.00				
1.00E-02	4	0	9	0	9	100.00				
1.00E-03	4	0	5	0	5	100.00	0.66667	-3.6667	4.64E+03	4.64E+04
1.00E-04	1	3	1	3	4	25.00				
1.00E-05	0	4	0	7	7	0.00				
1.00E-06	0	4	0	11	11	0.00				
1.00E-07	0	4	0	15	15	0.00				
1.00E-08	0	4	0	19	19	0.00				

PBS rep3

Dilution	pserved CPE and I(		Cumulative value		Total	6 Positive	PD	LogID50	D50/volu	TCID50/ml
	#Positive	#Negative	↑ Positiv	↓ Negativ						
5.00E-01	4	0	16	0	16	100.00				
1.00E-01	4	0	12	0	12	100.00				
1.00E-02	4	0	8	0	8	100.00				
1.00E-03	3	1	4	1	5	80.00	0.5	-3.5	3.16E+03	3.16E+04
1.00E-04	1	3	1	4	5	20.00				
1.00E-05	0	4	0	8	8	0.00				
1.00E-06	0	4	0	12	12	0.00				
1.00E-07	0	4	0	16	16	0.00				
1.00E-08	0	4	0	20	20	0.00				

This is for ten-fold dilution.

This TCID50 is the proportion with virus volume in the experiment.

Innoculated virus(volume)/we 0.1 ml

Direction: Fill in the cells which are light blue.

SPI rep1

Dilution	pserved CPE and I(		Cumulative value		Total	6 Positive	PD	LogID50	D50/volu	TCID50/ml
	#Positive	#Negative	↑ Positiv	↓ Negativ						
5.00E-01	0	4	0	4	4	0.00				
1.00E-01	0	4	0	8	8	0.00				
1.00E-02	0	4	0	12	12	0.00				
1.00E-03	0	4	0	16	16	0.00				
1.00E-04	0	4	0	20	20	0.00				
1.00E-05	0	4	0	24	24	0.00				
1.00E-06	0	4	0	28	28	0.00				
1.00E-07	0	4	0	32	32	0.00				
1.00E-08	0	4	0	36	36	0.00				

SPI rep2

Dilution	pserved CPE and I(		Cumulative value		Total	6 Positive	PD	LogID50	D50/volu	TCID50/ml
	#Positive	#Negative	↑ Positiv	↓ Negativ						
5.00E-01	0	4	0	4	4	0.00				
1.00E-01	0	4	0	8	8	0.00				
1.00E-02	0	4	0	12	12	0.00				
1.00E-03	0	4	0	16	16	0.00				
1.00E-04	0	4	0	20	20	0.00				
1.00E-05	0	4	0	24	24	0.00				
1.00E-06	0	4	0	28	28	0.00				
1.00E-07	0	4	0	32	32	0.00				
1.00E-08	0	4	0	36	36	0.00				

SPI rep3

Dilution	pserved CPE and I(		Cumulative value		Total	6 Positive	PD	LogID50	D50/volu	TCID50/ml
	#Positive	#Negative	↑ Positiv	↓ Negativ						
5.00E-01	0	4	0	4	4	0.00				
1.00E-01	0	4	0	8	8	0.00				
1.00E-02	0	4	0	12	12	0.00				
1.00E-03	0	4	0	16	16	0.00				
1.00E-04	0	4	0	20	20	0.00				
1.00E-05	0	4	0	24	24	0.00				
1.00E-06	0	4	0	28	28	0.00				
1.00E-07	0	4	0	32	32	0.00				
1.00E-08	0	4	0	36	36	0.00				

This is for ten-fold dilution.

This TCID50 is the proportion with virus volume in the experiment.

Innoculated virus(volume)/we 0.1 ml
